# Robust meta gradient learning for high-dimensional data with noisy-label ignorance

**DOI:** 10.1371/journal.pone.0295678

**Published:** 2023-12-11

**Authors:** Ben Liu, Yu Lin

**Affiliations:** School of Statistics, Southwestern University of Finance and Economics, Chengdu, Sichuan, China; Amrita Vishwa Vidyapeetham, INDIA

## Abstract

Large datasets with noisy labels and high dimensions have become increasingly prevalent in industry. These datasets often contain errors or inconsistencies in the assigned labels and introduce a vast number of predictive variables. Such issues frequently arise in real-world scenarios due to uncertainties or human errors during data collection and annotation processes. The presence of noisy labels and high dimensions can significantly impair the generalization ability and accuracy of trained models. To address the above issues, we introduce a simple-structured penalized *γ*-divergence model and a novel meta-gradient correction algorithm and establish the foundations of these two modules based on rigorous theoretical proofs. Finally, comprehensive experiments are conducted to validate their effectiveness in detecting noisy labels and mitigating the curse of dimensionality and suggest that our proposed model and algorithm can achieve promising outcomes. Moreover, we open-source our codes and distinctive datasets on GitHub (refer to https://github.com/DebtVC2022/Robust_Learning_with_MGC).

## Introduction

Large datasets in industry, characterized by noisy labels and high dimensions, are increasingly common [1–9]. The labels are often generated through unknown processes or manual annotation, leading to noisy labels [10, 11]. This, in turn, reduces the robustness of models and increases learning costs [12], negatively impacting model generalization and accuracy [13]. Recent research [1, 3, 5, 10–12, 14–16] has focused on developing strategies to train robust models using such datasets or effectively eliminate noisy labels.

Data-driven methods [1, 5, 11] address noisy labels by assuming the Classification Noise Process (CNP) [17] and using data distribution and information to pre-filter the labels. Model-driven studies [7, 10, 14–16], on the other hand, focus on fitting robust models to noisy datasets to learn the true labels. Some studies [7, 11, 18] have improved these approaches by incorporating concepts from curriculum learning, resistance learning, and peer networks to correct noisy labels.

However, most of these studies have primarily focused on optimizing network structures, resulting in longer training times and higher costs. The *γ*-divergence, initially introduced as the “density power divergence of type-zero” [19], is a loss function that ensures model robustness by comparing two probability density functions [16]. Unfortunately, when modeling large datasets in the industry using *γ*-divergence, it often leads to unsatisfactory results due to the introduction of numerous predictive variables to mitigate modeling biases (known as the Curse of Dimensionality) [20]. To address this issue, we propose a simple-structured penalized *γ*-divergence model and a meta gradient correction optimization algorithm to robustly model noisy labels and high-dimensional datasets. We instantiate our approach by deriving the model in the specific scenario of logistic regression and then extend it to the framework of generalized linear models.

Concretely, we begin by defining the *γ*-divergence and introducing a class of penalty function $P_{\lambda_{n}}\left(\cdot \right)$ mentioned in Liu et al. (2023) [21], which yields the objective function Eq (9). Based on this, we derive the consistency and efficiency of our model parameter estimation (refer to Theorems 1 and 2), as well as the empirical risk upper bound of our model (refer to Theorem 3). Furthermore, since the weight function of *γ*-divergence $\omega_{i}={\left[\frac{\text{exp}\{Y_{i}\left(\gamma +1\right)\beta^{T}X_{i}\}}{1+\text{exp}\{\left(\gamma +1\right)\beta^{T}X_{i}\}}\right]}^{\frac{\gamma }{\gamma +1}}$ of (*X*_*i*_, *Y*_*i*_) shows the possibility whether *Y*_*i*_ is a noisy label, we propose a meta gradient correction algorithm based on *ω*_*i*_ and establish its theoretical basis (refer to Theorem 4). This algorithm is a variant of stochastic gradient descent [22], which achieves effective discrimination between noisy label samples and non-noisy label samples by setting a pre-defined threshold. Finally, we extend our theoretical results to demonstrate the generalizability of our approach to a wider range of cases.

This paper makes three key contributions to prior work on finding, understanding, and learning with noisy labels,

- We propose a simple-structured penalized *γ*-divergence model and lay its foundations on theoretical proofs that effectively reduce manual feature engineering and improve modeling efficiency.
- We propose a novel meta gradient correction algorithm, and demonstrate its effectiveness through solid theoretical proofs and rich experiments that enable it to detect noisy labels and reduce the cost of manual labeling.
- We open-source our experimental code and data, showcasing the promising outcomes of our model on two tasks: detecting label errors, and learning from noisy labels.

## Related works

### Noisy label

Many studies focus on how to detect, discover, and recognize noisy labels from industrial datasets, which are mainly divided into method for reweighting examples [23–27], method for estimating the noise transition matrix [28–32], method for optimizing gradient descent and training procedures [10, 33, 34], method for selecting confident examples [1, 5, 35, 36], method for introducing regularization [4, 7, 37–39], method for designing robust loss functions [8, 9, 14, 16, 40–44], and method for generating pseudo labels [45–49].

In addition, some advanced start-of-the-art methods combine several techniques, e.g., MentorNet [11], DivideMix [50], Iterative Learning [51], and ELR+ [52].

### *γ*-divergence

The *γ*-divergence, which is first introduced in [19] with the name density power divergence of type-zero, is defined for two probability density functions. *γ*-divergence is closely related to other divergence measures, such as Kullback-Leibler (KL) divergence, but includes a tunable parameter *γ* that allows for more flexibility in the metric. Fujisawa and Eguchi (2008) [53] introduced *γ*-divergence later. Hung et al. (2018) [14] proposed a robust mislabeled logistic regression model based on the original form of *γ*-divergence defined by Jones et al. (2001) [19], called the *γ*-logistic regression.

### Penalized empirical likelihood

Penalized Empirical Likelihood has evolved as an essential statistical inference method, addressing challenges in small sample settings with nonparametric and semiparametric modeling. The origins of the method can be traced back to Owen (1988) [54], who introduced the concept of empirical likelihood, a nonparametric likelihood-based method for estimating unknown parameters in statistical models. In 1996, Qin and Lawless [55] further developed the theory and proved its efficiency and adaptability in various scenarios.

The incorporation of penalization in empirical likelihood was first introduced by Fan and Li (2001) [56]. They demonstrated how to use penalized empirical likelihood in variable selection and model estimation in high-dimensional and nonparametric settings. Later, Chen and Pouzo (2009) [57] proposed the usage of L1-penalty in empirical likelihood estimation, offering a more robust framework for handling high-dimensional data. Furthermore, MCP was initially proposed by Zhang (2010) [58] to overcome the drawbacks of the LASSO penalty, such as bias and lack of sparsity.

In recent years, the application of penalized empirical likelihood has expanded into various fields. Two exciting applications of this method are found in Fan et al. (2016) [59] and Shi et al. (2016) [60], where they employed a penalized empirical likelihood method for variable selection in high-dimensional settings.

## Methodology

This section proposes a novel noisy-label-ignore *γ*-divergence model for high-dimensional data with solid theoretical foundations. We substitute logistic regression as a specific case into this framework for a more explicit description. We first give some notations and mathematical derivation of the optimization objective for *γ*-divergence. Then, we propose the *γ*-divergence model by introducing a penalty function and proving the parameter consistency, asymptotic normality, and optimal risk upper bound for this model. Finally, we extend this model to the general case and introduce our meta gradient correction algorithm for implementing robust learning for noisy labels.

### *γ*-divergence and its optimization objective

In the binary classification problem, let X be the sample space, *Y*_0_ be the true binary response taking values in {0, 1}, and *X* be the *d*-dimensional vector of covariates. Let *P*_(*X*, *Y*_0_)_ and *P*_*X*_ denote the joint distribution of (*X*, *Y*_0_) and the distribution of *X*, respectively. Let the conditional success probability of *Y*_0_ be $\eta \left(x\right)=P\left(Y_{0}=1|X=x\right)$. In many real applications, we can only observe response *Y* contaminated with noise instead of the true response *Y*_0_. Define the mislabeled probability

$$\begin{array}{c} \tau_{01}\left(x\right)=P\left(Y=1|Y_{0}=0,X=x\right), \end{array}$$ (1)

and

$$\begin{array}{c} \tau_{10}\left(x\right)=P\left(Y=0|Y_{0}=1,X=x\right). \end{array}$$ (2)

Therefore, the conditional success probability of *Y*, which is denoted by

$$\begin{array}{c} \tilde{\eta }\left(x\right)=P\left\{Y=1|X=x\right\}, \end{array}$$ (3)

can be expressed by

$$\begin{array}{c} \begin{array}{cc} \tilde{\eta }\left(x\right) & =\left\{1-\tau_{10}\left(x\right)\right\}\eta \left(x\right)+\tau_{01}\left(x\right)\left\{1-\eta \left(x\right)\right\}\\ & =\left\{1-\tau_{01}\left(x\right)-\tau_{10}\left(x\right)\right\}\eta \left(x\right)+\tau_{01}\left(x\right). \end{array} \end{array}$$ (4)

Suppose that we observe independent and identical data pairs (*X*_*i*_, *Y*_*i*_), *i* = 1, …, *n*, with joint distribution *P*_(*X*, *Y*)_. The goal is to predict the true label *Y*_0_ of a new observation with covariates *X* using the model fitted by the data (*X*_1_, *Y*_1_), …, (*X*_*n*_, *Y*_*n*_).

Let *g*(*y*|*x*) be the underlying conditional probability density function of *Y*. By the definition of $\tilde{\eta }\left(x\right)$, we have $g\left(y|x\right)=\tilde{\eta }{\left(x\right)}^{y}{\left\{1-\tilde{\eta }\left(x\right)\right\}}^{1-y}$. Let *f*(*y*|*x*; ***β***) be the parametric conditional probability density function with the parameter $\beta \in R^{d}$, where *d* is the dimensions of ***β***. The *γ*-divergence between *g*(*y*|*x*) and *f*(*y*|*x*; ***β***) is defined to be

$$\begin{array}{c} \begin{array}{c} D_{\gamma }\left(g\left(\cdot |x\right),f\left(\cdot |x;\beta \right)\right)=\frac{1}{\gamma (\gamma +1)}[{∥g\left(\cdot |x\right)∥}_{\gamma +1}-\int {\left\{\frac{f(y|x;\beta )}{{∥f\left(\cdot |x;\beta \right)∥}_{\gamma +1}}\right\}}^{\gamma }g\left(y|x\right)\;dy], \end{array} \end{array}$$ (5)

where ${∥g\left(\cdot |x\right)∥}_{\gamma +1}={\left\{\int g{\left(y|x\right)}^{\gamma +1}\;dy\right\}}^{\frac{1}{\gamma +1}}$. Suppose that there exists a true parameter value $\beta_{0}={\left(\beta_{10},...,\beta_{d0}\right)}^{T}={\left(\beta_{10}^{T},\beta_{20}^{T}\right)}^{T}$ satisfying ***β***_20_ = 0, where $\beta_{10}\in R^{s\times 1}$, $\beta_{20}\in R^{(d-s)\times 1}$, and *s* is a finite constant, such that *g*(*y*|*x*) = *f*(*y*|*x*; ***β***_0_). By Theorem 3.1 in Fujisawa and Eguchi (2008) [53], *D*_*γ*_{*g*(⋅|*x*), *f*(⋅|*x*; ***β***_0_)} = 0. Therefore, ***β*** can be estimated by minimizing *D*_*γ*_{*g*(⋅|*x*), *f*(⋅|*x*; ***β***)}. To eliminate the randomness of *X*, we take expectations with respect to *X* in (5) and have

$$\begin{array}{c} \begin{array}{c} E_{X}\left[D_{\gamma }\left(g\left(\cdot |X\right),f\left(\cdot |X;\beta \right)\right)\right]=\frac{1}{\gamma (\gamma +1)}[E_{X}{\{∥g\left(\cdot |X\right)∥}_{\gamma +1}\}-E_{X,Y}[{\left\{\frac{f(Y|X;\beta )}{{∥f\left(\cdot |X;\beta \right)∥}_{\gamma +1}}\right\}}^{\gamma }]]\;. \end{array} \end{array}$$

The *γ*-logistic regression estimates ***β*** by minimizing $E_{X}\left[D_{\gamma }\left(g\left(\cdot |X\right),f\left(\cdot |X;\beta \right)\right)\right]$. Since $E_{X}{\{∥g\left(\cdot |X\right)∥}_{\gamma +1}\}$ is a constant, we have

$$\begin{array}{c} \begin{array}{c} \text{arg}\underset{\beta }{\text{min}}\;E_{X}\left[D_{\gamma }\left(g\left(\cdot |X\right),f\left(\cdot |X;\beta \right)\right)\right]=\text{arg}\underset{\beta }{\text{max}}E_{X,Y}[{\left\{\frac{f(Y|X;\beta )}{{∥f\left(\cdot |X;\beta \right)∥}_{\gamma +1}}\right\}}^{\gamma }]\;, \end{array} \end{array}$$ (6)

where $E_{X}$ and $E_{X,Y}$ denote the expectation with respect to *X* and (*X*, *Y*), respectively.

In particular, logistic regression assumes that

$$\begin{array}{c} \begin{array}{c} f\left(y|x;\beta \right)=\pi {\left(x;\beta \right)}^{y}{\left\{1-\pi \left(x;\beta \right)\right\}}^{1-y}, \end{array} \end{array}$$ (7)

where $\pi \left(x;\beta \right)=\frac{\text{exp}(\beta^{T}x)}{1+\text{exp}(\beta^{T}x)}$. Given the observations (*X*_1_, *Y*_1_), …, (*X*_*n*_, *Y*_*n*_), and substituting (7) into (6), the estimator $\hat{\beta }$ is defined by

$$\begin{array}{c} \hat{\beta }=\text{arg}\underset{\beta }{\text{max}}\frac{1}{n}\sum_{i=1}^{n}{\left[\frac{\text{exp}\{Y_{i}\left(\gamma +1\right)\beta^{T}X_{i}\}}{1+\text{exp}\{\left(\gamma +1\right)\beta^{T}X_{i}\}}\right]}^{\frac{\gamma }{\gamma +1}}\;. \end{array}$$ (8)

### Penalized *γ*-divergence model and its oracle properties

As claimed by Fujisawa and Eguchi (2008) [53] and Kawashima and Fujisawa (2017) [16], *γ*-divergence is still very robust to noisy data. However, when the dimension *d* of the covariate *X* diverges, it can be shown that the estimator in (8) is no longer consistent with the true parameter ***β***_0_, and the estimation equation is no longer robust [16], which leads to the model prediction will have serious error accumulation and reduces the prediction accuracy [16, 61]. Hence, to solve these problems, we introduce a class P of penalty function $P_{\lambda_{n}}\left(\cdot \right)$ mentioned in Liu et al. (2023) [21] as follows,

$$\begin{array}{c} \begin{array}{cc} \;\;\;\;\;\;P & =\{P_{\lambda_{n}}\left(t\right):P_{\lambda_{n}}\left(t\right)\;is\;increasing\;in\;t\in \left[0,\infty \right)\;and\;has\;continuous\;derivative\\ & \;\;\;\;\;\;\;\;P_{\lambda_{n}}^{{}^{'}}\left(t\right)\;for\;any\;t\in \left(0,\infty \right)\;with\;P_{\lambda_{n}}^{{}^{'}}\left(t\right)\in \left(0,\infty \right)\;and\;P_{\lambda_{n}}\left(0\right)=0\}\;. \end{array} \end{array}$$

Then the objective function of the penalized *γ*-logistic regression is

$$\begin{array}{c} Q\left(\beta \right)=\sum_{i=1}^{n}{\left[\frac{\text{exp}\{Y_{i}\left(\gamma +1\right)\beta^{T}X_{i}\}}{1+\text{exp}\{\left(\gamma +1\right)\beta^{T}X_{i}\}}\right]}^{\frac{\gamma }{\gamma +1}}-n\sum_{j=1}^{d}P_{\lambda_{n}}\left(\right|\beta_{j}\left|\right), \end{array}$$ (9)

where $P_{\lambda_{n}}$ is a penalty function in class P with tuning parameter λ_*n*_, and ***β***_*j*_, *j* = 1, …, *d*, is an element in the vector *β*. Hence, the estimator ${\hat{\beta }}_{pen}$ is defined by ${\hat{\beta }}_{pen}=\text{arg}{\text{max}}_{\beta }Q\left(\beta \right)$.

We still need consistent results here since we focus on a parametric model. We first show two regularity conditions in Lemmas 1 and 2 and then give the consistency and asymptotic normality of parameter estimation in Theorems 1 and 2.

**Lemma 1** *(Regularity Condition)*. *Let V*_*i*_ = (*X*_*i*_, *Y*_*i*_) *and*

$$\begin{array}{c} Q\left(\beta \right)=L\left(\beta \right)-n\sum_{j=1}^{d}P_{\lambda_{n}}\left(\right|\beta_{j}\left|\right), \end{array}$$ (10)

*where*

$$\begin{array}{c} L\left(\beta \right)=\sum_{i=1}^{n}\frac{{\left[{\left\{\pi \left(X_{i};\beta \right)\right\}}^{Y_{i}}{\left\{1-\pi \left(X_{i};\beta \right)\right\}}^{1-Y_{i}}\right]}^{\gamma }}{{\left[{\left\{\pi \left(X_{i};\beta \right)\right\}}^{\gamma +1}+{\left\{1-\pi \left(X_{i};\beta \right)\right\}}^{\gamma +1}\right]}^{\frac{\gamma }{\gamma +1}}}, \end{array}$$ (11)

*and* *β*_*j*_, *j* = 1, …, *d*, *is an element in the vector* ***β***.

*Let P*(*Y*_*i*_ = 1) *be the probability that the sample label is 1 and*

$$\begin{array}{c} P\left(Y_{i}=0\right)+P\left(Y_{i}=1\right)=1, \end{array}$$ (12)

*then the first derivatives of L*(***β***) *satisfying the equations*

$$\begin{array}{c} E_{Y}(\frac{\partial \phi (\beta )}{\partial \beta })=0, \end{array}$$ (13)

*at* ***β*** = ***β***_0_, *where* $\phi \left(\beta \right)=\frac{{\left[{\left\{\pi \left(X_{i};\beta \right)\right\}}^{Y_{i}}{\left\{1-\pi \left(X_{i};\beta \right)\right\}}^{1-Y_{i}}\right]}^{\gamma }}{{\left[{\left\{\pi \left(X_{i};\beta \right)\right\}}^{\gamma +1}+{\left\{1-\pi \left(X_{i};\beta \right)\right\}}^{\gamma +1}\right]}^{\frac{\gamma }{\gamma +1}}}$.

**Lemma 2** *(Regularity Condition). The Fisher information matrix*,

$$\begin{array}{c} I\left(\beta \right)=E[\{\frac{\partial \phi (\beta )}{\partial \beta }\}{\left\{\frac{\partial \phi (\beta )}{\partial \beta }\right\}}^{T}], \end{array}$$ (14)

*is finite and positive definite at* ***β*** = ***β***_0_.

**Theorem 1** *(Consistency and Convergence Rate). Let V*_*i*_ = (*X*_*i*_, *Y*_*i*_) *be independent and identically distributed and* $P_{\lambda_{n}}$ *be a penalty function in class* P *with tuning parameter* λ_*n*_. *Let*

$$\begin{array}{c} a_{n}=max\{P_{\lambda_{n}}^{{}^{'}}\left(\right|\beta_{j0}|):\beta_{j0}\neq 0\}, \end{array}$$ (15)

*and*

$$\begin{array}{c} b_{n}=max\{|P_{\lambda_{n}}^{{}^{''}}\left(\right|\beta_{j0}|)|:\beta_{j0}\neq 0\}. \end{array}$$ (16)

*Assume that* $a_{n}=o_{p}\left(n^{-\frac{1}{2}}\right)$ *and* $b_{n}=o_{p}\left(1\right)$ , *there is a local maximizer* $\hat{\beta }$ *of* Q(β) *such that* $\left|\right|\hat{\beta }-\beta_{0}\left|\right|=O_{p}\left(n^{-\frac{1}{2}}+a_{n}\right)$ *under some regularity conditions*.

It is clear from Theorem 1 that by choosing a proper $P_{\lambda_{n}}$, and there exists a root-n consistent penalized likelihood estimator $\hat{\beta }$. Let *I*(***β***_0_) be the Fisher information matrix and let *I*_1_(***β***_10_) be the Fisher information knowing ***β***_20_ = 0. We now show that the estimator $\hat{\beta }$ possesses the sparsity property $\hat{\beta_{2}}=0$ and the asymptotic normality, which is also known as the Oracle property [56].

**Theorem 2** *Let* $\hat{\beta }={\left(\hat{\beta_{1}},\hat{\beta_{2}}\right)}^{T}$ *be a local maximizer of* Q(β) *in Theorem 1*, *V*_*i*_ = {*X*_*i*_, *Y*_*i*_} *be independent and identically distributed and* $P_{\lambda_{n}}$ *be a penalty function in class* P *with tuning parameter* λ_*n*_. *Let* $a_{n}=max\{P_{\lambda_{n}}^{{}^{'}}\left(\right|\beta_{j0}|):\beta_{j0}\neq 0\}$ *where assuming* $a_{n}=o_{p}\left(n^{-\frac{1}{2}}\right)$. *Assume that* $n^{\frac{1}{4}}\lambda_{n}\to \infty \;when\;n\to \infty$ *and* λ_*n*_ → 0, *then for any constant C, under some regularity conditions*, $\hat{\beta }$ *satisfy*:

*(a)Sparsity:* $\hat{\beta_{2}}=0$ ,

*(b)Asymptotic Normality:*

$$\begin{array}{c} \sqrt{n}{\left(I_{1}\left(\beta_{10}\right)+\Sigma \right)}^{-1}\left\{\hat{\beta_{1}}-\beta_{10}+{\left(I_{1}\left(\beta_{10}\right)+\Sigma \right)}^{-1}b\right\}\to \;N\left(0,I_{1}\left(\beta_{10}\right)\right), \end{array}$$ (17)

*where*

$$\begin{array}{c} b=\{P_{\lambda_{n}}^{{}^{'}}\left(\right|\beta_{10}|)sgn\left(\beta_{10}\right),...,P_{\lambda_{n}}^{{}^{'}}\left(\right|\beta_{s0}|)sgn\left(\beta_{s0}\right){\}}^{T}, \end{array}$$ (18)

*and*

$$\begin{array}{c} \Sigma =\text{diag}\{P_{\lambda_{n}}^{{}^{''}}\left(\right|\beta_{10}|),...,P_{\lambda_{n}}^{{}^{''}}\left(\right|\beta_{s0}|)\}. \end{array}$$ (19)

Theorems 1 and 2 reveal that $\hat{\beta }$ possesses consistency and asymptotic normality. Moreover, as claimed by Cannings et al. (2020) [62], a model trained on noisy labels should be close to the optimal Bayes model under some certain conditions. Hence, we aim to get the bound of excess risk between our model and the optimal Bayes model. First, we need some notations. Let *C*(*x*) be the predicted label of {0, 1} value and given *x*, define

$$\begin{array}{c} C\left(x\right)=\{\begin{array}{cc} 1,\; & f\left(x\right)\geq \frac{1}{2}\;,\\ 0,\; & f\left(x\right)<\frac{1}{2}\;, \end{array} \end{array}$$ (20)

and $\epsilon =||f\left(x\right)-\tilde{\eta }\left(x\right){\left|\right|}_{\infty }$, which is the estimation error between the predicted label by *f*(*x*) and the noisy conditional probability $\tilde{\eta }\left(x\right)$. We give the following Definitions 1 and 2, for the Bayes classifier.

**Definition 1**. *Given x*, *the optimal classifier under clean labels is Bayes classifier, denoted as* C*(*x*), *where*

$$\begin{array}{c} C^{*}\left(x\right)=\{\begin{array}{cc} 1,\; & \eta \left(x\right)\geq \frac{1}{2}\;,\\ 0,\; & \eta \left(x\right)<\frac{1}{2}\;. \end{array} \end{array}$$ (21)

**Definition 2**. *Given x*, *the optimal classifier under noisy labels is Bayes classifier, denoted as* ${\tilde{C}}^{*}\left(x\right)$, *where*

$$\begin{array}{c} {\tilde{C}}^{*}\left(x\right)=\{\begin{array}{cc} 1,\; & \tilde{\eta }\left(x\right)\geq \frac{1}{2}\;,\\ 0,\; & \tilde{\eta }\left(x\right)<\frac{1}{2}\;. \end{array} \end{array}$$ (22)

Definitions 1 and 2 tell us that the Bayes classifier minimizes the classification risk under noisy labels. Furthermore, we then introduce the Tsybakov Condition by following Assumption 1, which is the basis of Lemma 3 and Theorem 3.

**Assumption 1**. *(Tsybakov Condition). There exist constants M*, λ ≥ 0 *and* $t_{0}\in \left(0,\frac{1}{2}\right]$, *such that for all* 0 ≤ *t* ≤ *t*_0_ *and x*, *the following inequality holds*,

$$\begin{array}{c} P(|\eta \left(x\right)-\frac{1}{2}\left|\leq t\right)\leq Mt^{\lambda }. \end{array}$$ (23)

This condition, also called margin assumption, stipulates that the uncertainty of *η*(*x*) is bounded. In other words, the margin region close to the decision boundary, {x∈X|η(x)=1/2}, has a bounded volume. Moreover, we define the subspace that the Tsybakov condition holds as $x\in S^{c}$, which means that for sufficiently small *t* ≤ *t*_0_, $S=\{x\in X:\frac{1}{2}-t\leq \eta \left(x\right)\leq \frac{1}{2}+t\}$ and $X=S\cup S^{c}$. Hence, let

$$\begin{array}{c} D=\{x\in S^{c}:{\tilde{C}}^{*}\left(x\right)=C^{*}\left(x\right)\}, \end{array}$$ (24)

and based on the Assumption 1, we can obtain Lemma 3 and Theorem 3.

Based on the above definitions and assumptions, Lemma 3 shows the bound of the model *R*_*our*_(*x*) and the Bayes classifier ${\tilde{R}}^{*}\left(x\right)$ under the noisy labels. And Theorem 3 is the main result of this paper, revealing that our model *R*_*our*_(*x*) and the optimal Bayes model R*(*x*) under the case of noisy labels still have an upper bound of excess risk.

**Lemma 3**. *(Upper bound of Excess Risk between R*_*our*_(*x*) *and* ${\tilde{R}}^{*}\left(x\right)$). *Assume η*(*x*) *statisfies the Tsybakov condition with constants M*, λ > 0, *and* $t_{0}\in \left(0,\frac{1}{2}\right]$. *Assume again that τ*_01_(*x*) + *τ*_10_(*x*) < 1. *Given x*, *for all* 0 ≤ *t* ≤ *t*_0_, *then the excess risk is*,

$$\begin{array}{c} R_{our}\left(x\right)-{\tilde{R}}^{*}\left(x\right)\leq M{\left\{O\left(\epsilon \right)\right\}}^{\lambda }, \end{array}$$ (25)

*as t* ≤ *t*_0_ → 0, *where*

$$\begin{array}{c} R_{our}\left(x\right)=\int_{X}P\left(Y_{0}\neq C\left(x\right)|X=x\right)dP_{X}\left(x\right), \end{array}$$ (26)

*and*

$$\begin{array}{c} {\tilde{R}}^{*}\left(x\right)=\int_{X}1\left\{Y_{0}\neq {\tilde{C}}^{*}\left(x\right)\right\}dP_{X}\left(x\right). \end{array}$$ (27)

**Theorem 3**. *(Upper bound of Excess Risk between R*_*our*_(*x*) and R*(*x*)). *Assume η*(*x*) *statisfies the Tsybakov condition with constants M*, λ > 0, and $t_{0}\in \left(0,\frac{1}{2}\right]$. *Assume again that τ*_01_(*x*) + *τ*_10_(*x*) < 1. *Given x*, *for all* 0 ≤ *t* ≤ *t*_0_, *then the excess risk is*,

$$\begin{array}{c} R_{our}\left(x\right)-R^{*}\left(x\right)\leq M{\left\{O\left(\epsilon \right)\right\}}^{\lambda }+K\left(\lambda \right), \end{array}$$ (28)

*as t* ≤ *t*_0_ → 0, *where*

$$\begin{array}{c} R_{our}\left(x\right)=\int_{X}P\left(Y_{0}\neq C\left(X\right)|X=x\right)dP_{X}\left(x\right),\;R^{*}\left(x\right)=\int_{X}1\left\{Y_{0}\neq C^{*}\left(X\right)\right\}dP_{X}\left(x\right), \end{array}$$ (29)

*and* $K\left(\lambda \right)=M{\left\{\frac{\frac{1}{2}\left(\tau_{10}\left(x\right)-\tau_{01}\left(x\right)\right)}{1-\tau_{01}\left(x\right)-\tau_{10}\left(x\right)}\right\}}^{\lambda }$ *is a small value depend on* λ.

Theorem 3 indicates that the risk bound between our model and the best Bayes classifier will be dominated by the model’s estimation error *ϵ*. If the estimation error *ϵ* is smaller, our model and the Bayes classifier are closer.

Furthermore, the estimation error refers to the difference between the estimated parameters of the model and the real parameters. We can obtain from the consistency of parameters the estimation error *ϵ* will tend to 0 as *γ* → ∞ and *n* → ∞, so our model will converges to the Bayes model in the case of large samples.

Specifically, from Theorems 1 and 2, we can get $\left|\right|\hat{\beta }-\beta_{0}\left|\right|\leq M^{{}^{'}}\left(n^{-\frac{1}{2}}+a_{n}\right)$ with constant $M^{{}^{'}}$, which leads to the fact that $\epsilon \leq M^{{}^{'}}\left(n^{-\frac{1}{2}}+a_{n}\right)$. Hence, from Theorem 3, we have $R_{our}\left(x\right)-R^{*}\left(x\right)\leq M{M^{{}^{'}}}^{\lambda }{\left\{O\left(n^{-\frac{1}{2}}\right)+o\left(n^{-\frac{1}{2}}\right)\right\}}^{\lambda }.$

Finally, we can easily generalize the conclusions of Theorems 1, 2, and 3 of *γ*-logistic regression to the generalized linear model (GLM) and obtain Corollary 1, as logistic regression is essentially a GLM. The proof of Corollary 1 is trivial and would not be repeated in our paper.

**Corollary 1**. *Under the regularity conditions 1 and 2 and Assumption 1, for each generalized linear model (such as the Probit model), we can obtain the same results as described in Theorems 1, 2, and 3: (i) consistency, (ii) asymptotic normality, and (iii) convergence of the model*.

### Meta gradient correction algorithm

Although we have proved in the above subsection that our proposed model possesses oracle properties even in the noisy-label case, recent study [10] has shown that machine learning models gradually remember individual data while adapting to the data distribution. Therefore, when facing noisy labels, all statistics or machine learning methods inevitably encounter the problem of reduced generalization ability, and it is necessary to eliminate the impact of single data as much as possible through methods such as early stopping or dropout. However, these methods passively eradicate the effect of a single data on model training. Excessive use of these methods often results in underfitting. Therefore, this paper proposes a regularized meta-learning algorithm, **Meta Gradient Correction (MGC)**, for noisy label data.

From Hung Hung et al. (2018) [14], the *γ*-divergence weight function of (*X*_*i*_, *Y*_*i*_),

$$\begin{array}{c} \omega_{i}={\left[\frac{\text{exp}\{Y_{i}\left(\gamma +1\right)\beta^{T}X_{i}\}}{1+\text{exp}\{\left(\gamma +1\right)\beta^{T}X_{i}\}}\right]}^{\frac{\gamma }{\gamma +1}}, \end{array}$$ (30)

shows the possibility of whether *Y*_*i*_ is a noisy label. In other words, if *ω*_*i*_ is larger, it means that (*X*_*i*_, *Y*_*i*_) has a more significant effect on model training and is less likely to be a noisy label. If *ω*_*i*_ is smaller, the sample Label *Y*_*i*_ is more likely to be a noisy label. On the other hand, the estimation criterion of minimum *γ*-divergence is to maximize the objective function *Q*(***β***), which leads to using the stochastic gradient ascent algorithm.

Hence, in this part, we propose an adaptive algorithm based on a meta approach that modifies the optimization algorithm. For example, if the corresponding value of the *γ*-divergence weight function *ω*_*i*_ of the (*X*_*i*_, *Y*_*i*_) is large, then gradient descent is performed to ***β***. In contrast, the gradient ascent is performed to eliminate the effect of suspected noisy labels on model parameter convergence. To illustrate the validity of this algorithm, we further show Theorem 4.

**Theorem 4**. *(Convergence of MGC algorithm). Let the number of samples for each batch be N*, *where the number of noisy labeled samples is N*_*noisy*_, *and the number of non-noisy labeled samples is N*_*non* − *noisy*_, *i.e., N* = *N*_*noisy*_ + *N*_*non*−*noisy*_. *Without loss of generality, assume that N*_*non*−*noisy*_ > *N*_*noisy*_. *Moreover, the function we want to minimize*, $Q\left(\beta \right):R^{N}\to R$, *is continuously differentiable, and we assume that Q*(***β***) *has Lipschitz continuous gradients with constant L, i.e., there exists a constant L* > 0 *such that*, $|\nabla Q\left(\beta_{1}\right)-\nabla Q\left(\beta_{2}\right)\left|\leq L\right|\beta_{1}-\beta_{2}|$ *for all* $\beta_{1},\;\beta_{2}\in R^{d}$. *This algorithm updates the gradient descent iteration as follows*, ***β***_*t*+1_ = ***β***_*t−η*_∇*Q*(***β***_*t*_), *where η* > 0 *is the learning rate. We then obtain that*,

$$\begin{array}{c} \begin{array}{c} Q\left(\beta_{t+1}\right)-Q\left(\beta_{t}\right)\leq -eta(N_{non-noisy}-N_{noisy})\{1-\frac{L\eta }{2}(N_{non-noisy}-N_{noisy})\}{\left|\nabla Q\left(\beta_{t}\right)\right|}^{2}, \end{array} \end{array}$$ (31)

*for all t* ≥ 0. *The function values Q*(***β***_*t*_) *form a non-increasing sequence, which implies that Q*(***β***_*t*_) *converges*.

Theorem 4 implicitly states that our proposed meta gradient correction algorithm makes our objective function *Q*(***β***) converge when the proportion of noisy labels is small (refer to *N*_*non*−*noisy*_ > *N*_*noisy*_). This is also consistent with the results shown in Section of Experiments and powerfully demonstrates the validity of our algorithm. Furthermore, following [21], we give three specific forms of the penalty function $P_{\lambda_{n}}\left(\cdot \right)$ as particular implementations of our algorithm. One is the SCAD penalty [56] where

$$\begin{array}{c} P_{SCAD}\left(\right|\beta_{j}\left|;\;\lambda_{n},\;\alpha \right)=\{\begin{array}{c} \lambda_{n}|\beta_{j}\left|,\;\;\;\;\;\;\;\;\;\;\;\;\;\;\;\;\right|\beta_{j}|\leq \lambda_{n}\;\\ -\frac{|\beta_{j}{|}^{2}-2\alpha \lambda_{n}\left|\beta_{j}\right|+\lambda_{n}^{2}}{2(\alpha -1)},\;\lambda_{n}\leq \left|\beta_{j}\right|\leq \alpha \\ \frac{{\left(\alpha +1\right)}^{2}\lambda_{n}}{2},\;\;\;\;\;\;\;\;\;\;\;\;\;\left|\beta_{j}\right|>\alpha , \end{array} \end{array}$$ (32)

where *β*_*j*_, *j* = 1, …, *d*, is an element in the vector *β*. Another one is the MCP [58] where $P_{MCP}\left(\right|\beta_{j}\left|;\lambda_{n},v\right)=\lambda \int_{0}^{|\beta_{j}|}{\left\{1-\frac{x}{\lambda v}\right\}}_{+}dx$, *j* = 1, …, *d*, to solve the problem of variable selection and estimation in high-dimensional case. The last one is the Lasso [63] where *P*_*Lasso*_(*β*_*j*_) = |*β*_*j*_|, *j* = 1, …, *d*. For ease of description and understanding, we substitute $\pi (X_{i};\beta )$ with logistic and probit models to illustrate our algorithm.

## Experiments

In this section, we evaluate the abilities of proposed model and algorithm, namely, the functionality of penalized *γ*-divergence model for learning high-dimensional data and the robustness of adaptive classification algorithm in modeling noisy data.

### Our dataset

We conducted numerous experiments on multiple simulation data and real data. These dataset has the following three characteristics.

- **Simulation data:** We generate noisy label dataset based on random seeds with three different sample dimensions and eight noisy label ratios. The training sample dimensions include: 200*500, 500*1000, and 1000*1500, and testing sample dimensions include: 100*500, 200*1000, and 200*1500. The noisy label ratios include 0.1, 0.2, 0.3, 0.4, 0.5, 0.6, 0.7, and 0.8.
- **Real data:** We obtain breast cancer data from the UCI Machine Learning Repository as real data (https://archive.ics.uci.edu/dataset/17/breast+cancer+wisconsin+diagnostic), which contains 571 samples and 31 feature variables. We randomly divide it into the training set and test set in a ratio of 0.8 and 0.2. The training set includes 456 samples, and the test set contains 115. We set the noisy label ratio in experiments to 0.1, 0.3, and 0.5. Following [18, 47, 52], we randomly select samples from the 0-labeled and 1-labeled samples in the training set (the number of samples chosen is the product of the noisy label ratio and the number of samples in this category). Finally, we flip the labels of the selected samples. Similarly, for the test set, we perform the same operation.
- Each noisy label dataset is used to train and test the effectiveness and capability of the eight models in learning and detecting noisy labels. The final test results for each model are based on the average of tests after 50 different training sessions.

### Settings of models

This subsection provides brief information about the experimental settings, and more details can be found in our open-sourced codes (https://github.com/DebtVC2022/Robust_Learning_with_MGC).

- **Baseline Models:** We select the logistic and probit models as baseline models following the research [14], with their optimal settings based on pre-experiments (described in the following paragraph).
- **Relevant Settings:** Our models contain multiple versions, obtained by combining two backbone models (refer to the logistic and probit models), three penalty functions (refer to the SCAD, MCP, and Lasso), and the proposed meta gradient correction algorithm. For all models, we select hyperparameters based on preliminary experiments and the prevalent findings [14, 18, 56, 61], and the final hyperparameters are set as follows: *γ* value of 0.5, λ value of 1, threshold value *Tv* of 0.5, learning rate *η* of 0.01.

### Evaluation metrics

Following the studies [14, 16], we choose the accuracy and F1-score to assess the ability of our model to model noisy data.

- **Accuracy based on contaminated label *Y*:** Following the studies [14, 16], we choose the accuracy to assess the ability of our model to learn noisy labels and then denote this evaluation metric as *ACC*_*noisy*, which records the number of correctly predicted data points among all data points (*X*, *Y*).
- **Accuracy based on true label *Y*_0_:** Following the studies [14, 16], we choose the accuracy to assess the ability of our model to learn noisy labels and then denote this evaluation metric as *ACC*_*true*, which records the number of correctly predicted data points among all data points (*X*, *Y*_0_).
- **Recall:** We denote this evaluation metric by *Recall*, which records the percentage of samples that a model correctly identifies as belonging to noisy label samples out of the total samples for that class.
- **Precision:** We denote this evaluation metric by *Precision*, which records the ratio of correctly identified noisy label samples to the total number of noisy label samples.
- **F1-score:** We denote this evaluation metric by *F*1, which is calculated by $\frac{2*Precision*Recall}{Precision+Recall}$.

These five metrics reflect the robustness of the model and its ability to detect noisy labels, and their larger values denote that the model has a stronger ability to model noisy labels. [14].

### Ablation study & analysis

In this subsection, we conduct comprehensive evaluations to demonstrate the effectiveness of our proposed model in learning with noisy labels. The evaluations are performed on simulated and real-world data by combining different backbone models (refer to two generalized linear models for binary classification problems: logistic regression and probit models), penalty functions (refer to SCAD [56], MCP [61], and Lasso [63]), and the proposed meta gradient correction (MGC) algorithm.

#### Analysis based on simulated data

**Fig 1:**
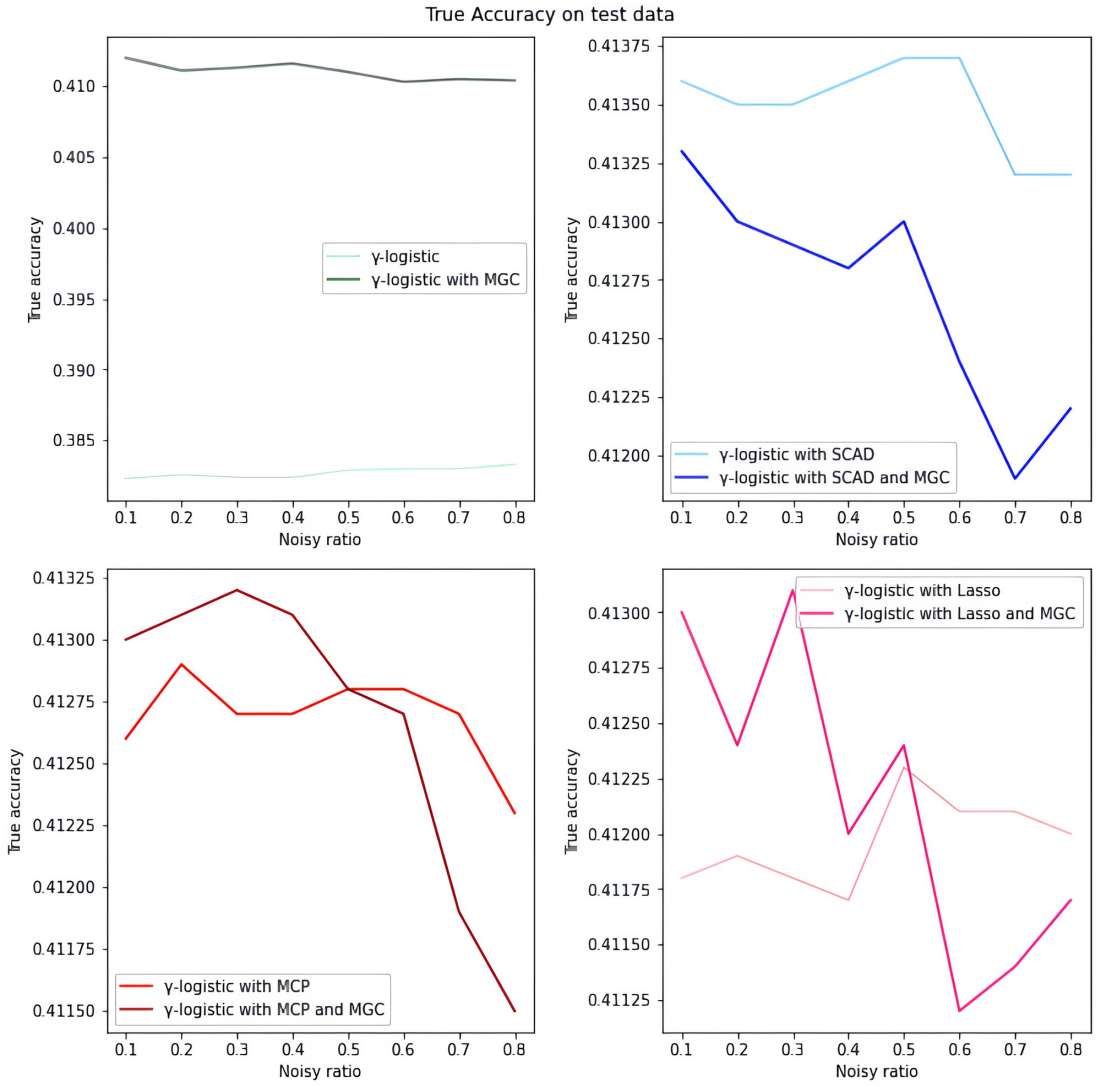
*ACC*_*true* on test data with *γ*-logistic model and 500*1000 training data size. When the proportion of noisy labels is small, introducing the meta gradient correction algorithm can improve the classification accuracy of the model, except when the meta gradient correction algorithm is combined with the SCAD penalty function.

**Fig 2:**
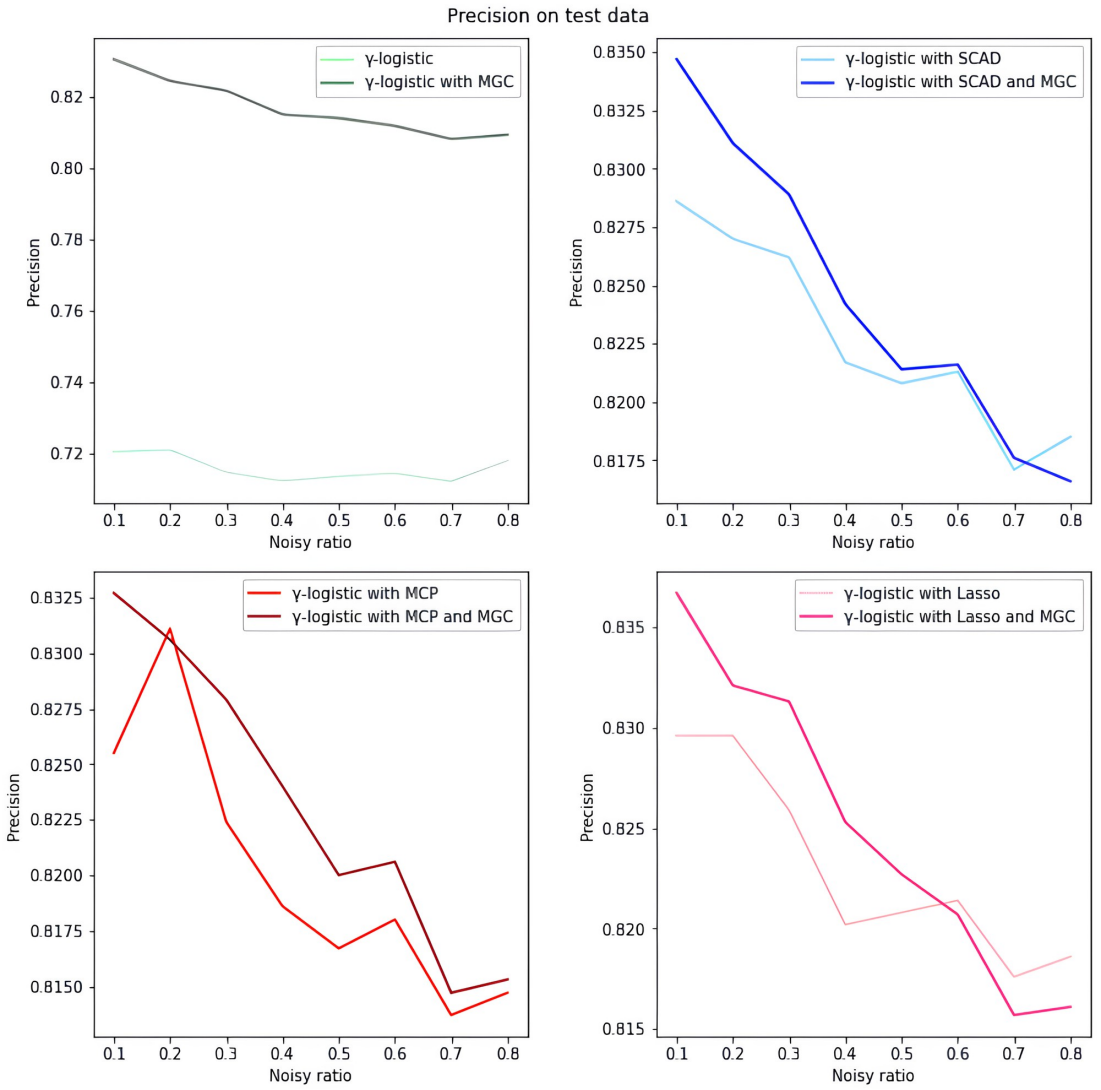
*Precision* on test data with *γ*-logistic model and 500*1000 training data size. All four subfigures show that the introduction of the meta gradient correction algorithm can increase the precision of model predictions, which means that the reliability of model predictions is improved.

**Fig 3:**
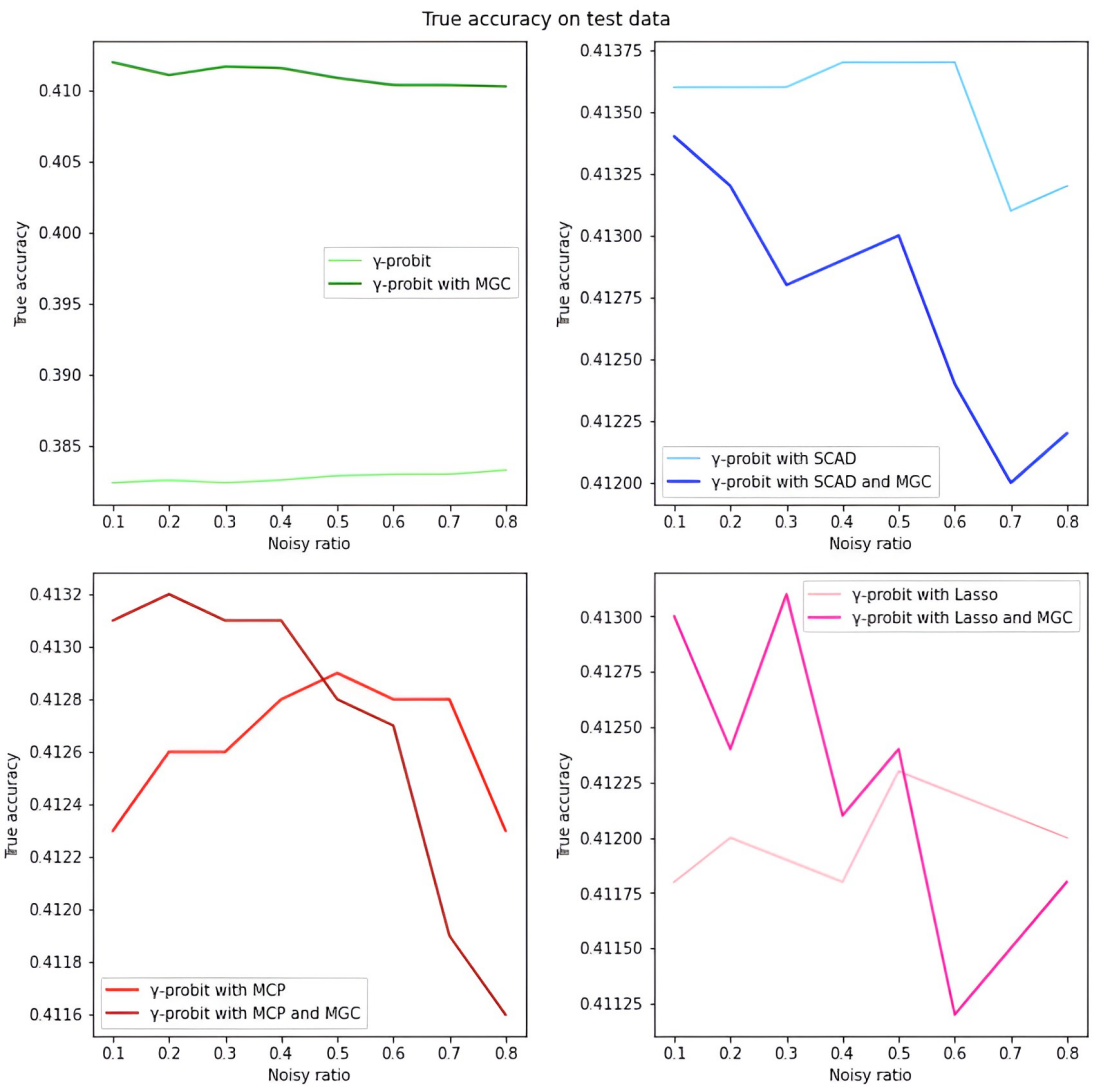
*ACC*_*true* on test data with *γ*-probit model and 500*1000 training data size. The four subfigures of this figure can lead to similar conclusions as in Fig 1.

**Fig 4:**
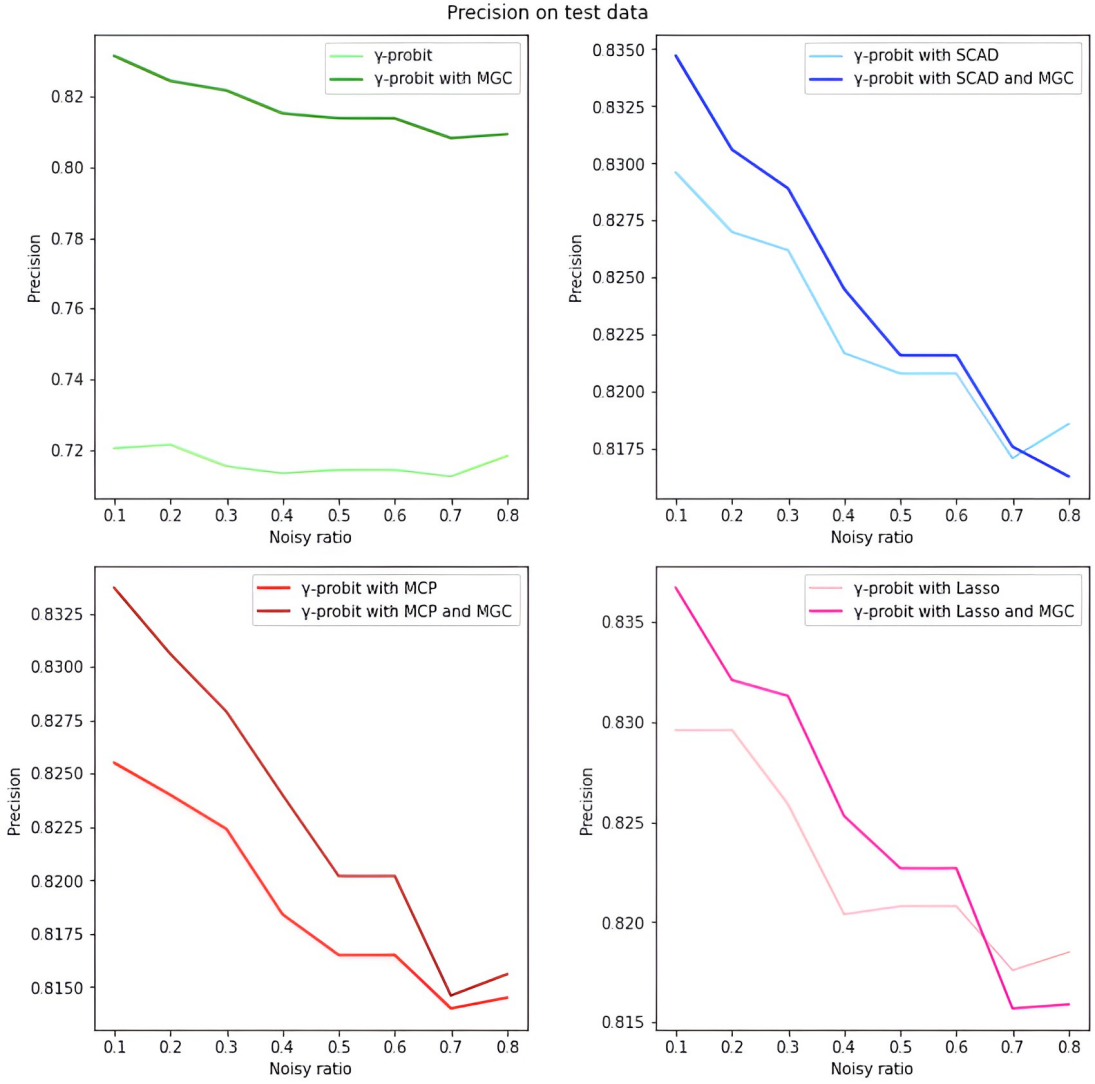
*Precision* on test data with *γ*-probit model and 500*1000 training data size. The four subfigures of this figure can lead to similar conclusions as in Fig 2.

**Fig 5:**
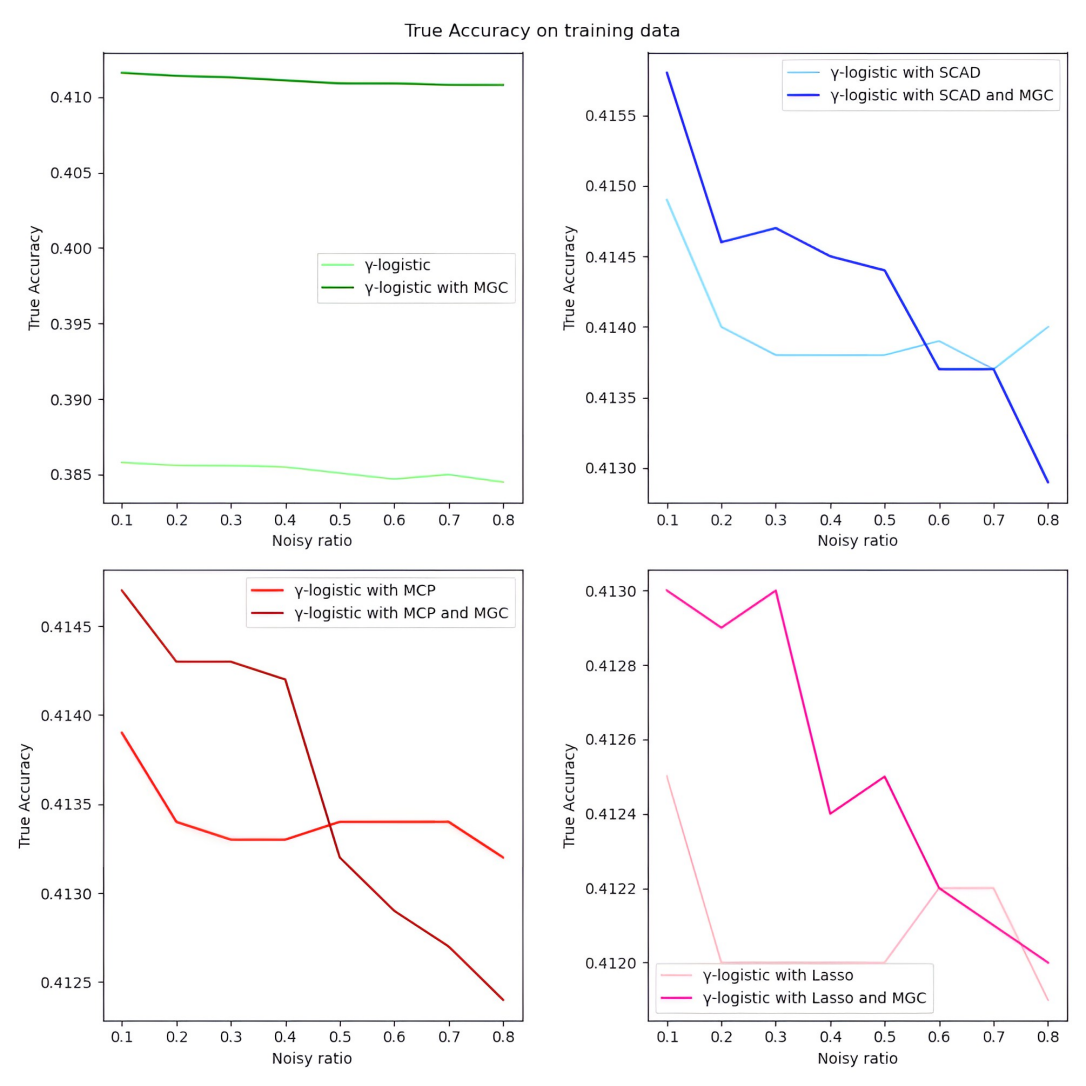
*ACC*_*true* on training data with *γ*-logistic model and 500*1000 training data size. When the proportion of noisy labels is small, introducing the meta gradient correction algorithm can improve the classification accuracy of the model.

**Fig 6:**
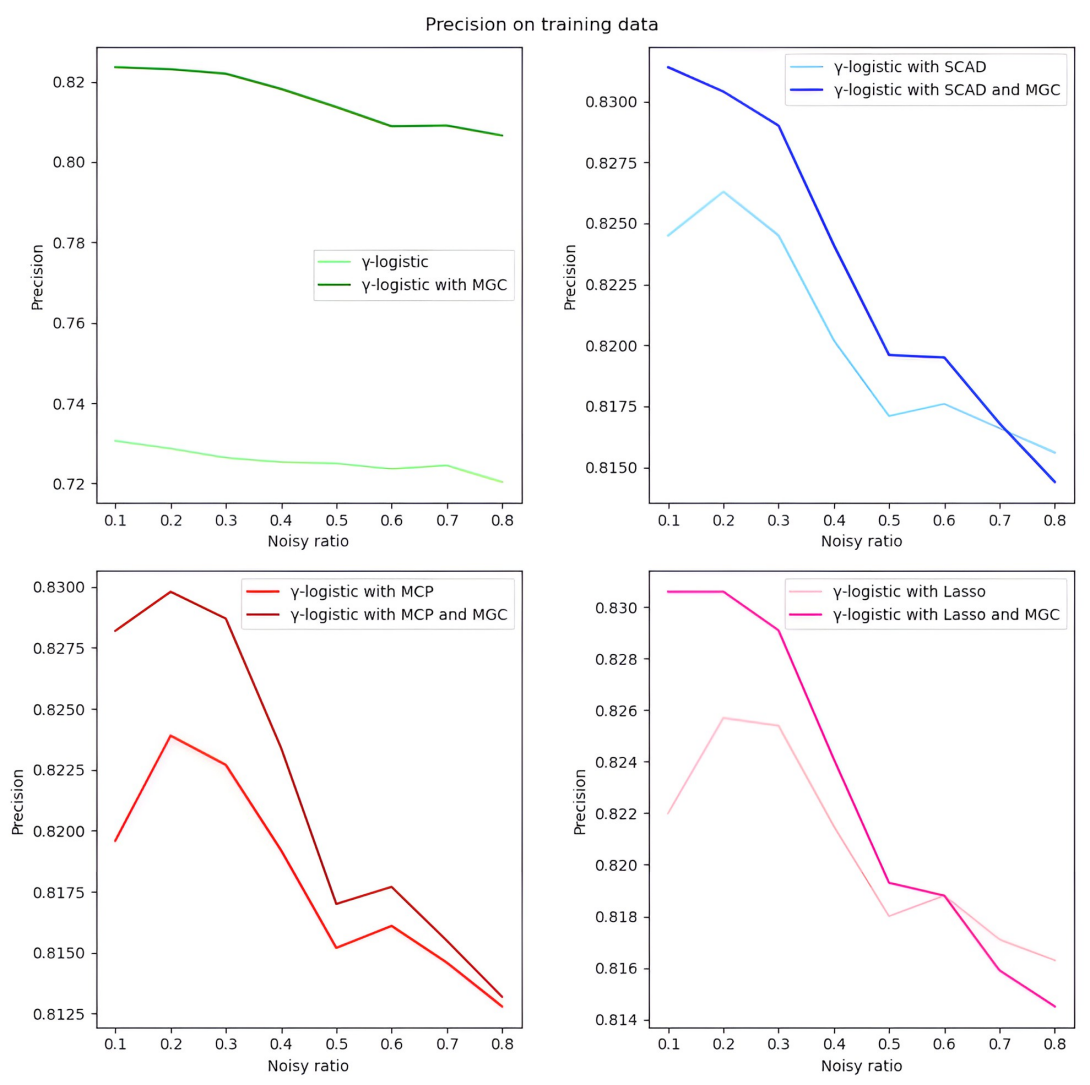
*Precision* on training data with *γ*-logistic model and 500*1000 training data size. All four subfigures show that the introduction of the meta gradient correction algorithm can increase the precision of model predictions, which means that the reliability of model predictions is improved.

*Overall analysis based on simulated data.* We first evaluate the model performance on simulated data under different settings. As illustrated in Tables 1–18, our proposed penalized *γ*-divergence model consistently achieves higher accuracy than the baseline model across different noisy ratios (refer to 0.1, 0.2, 0.3), backbone models, and penalty functions. This indicates the robustness of our model against noisy labels. More importantly, our model substantially improves *Recall*, *Precision*, and *F*1, the critical metrics for noisy label identification [62]. The results strongly verify the capability of our model in detecting and rectifying noisy labeled instances. In addition, in practical noisy label scenarios, we are more concerned with how similar the predicted labels $\hat{Y}$ are to the correct labels *Y*_0_ and what percentage of all noisy labels in the dataset are identified [62]. Hence, as depicted in Figs 1–6, we also display the trends of *ACC*_*true* and *Precision* under the different noisy ratios (refer to 0.1, 0.2, 0.3, 0.4, 0.5, 0.6, 0.7, 0.8).

**Table 1:** Results of evaulate metrics on the test data and 0.1 noisy ratio.

	ACC_noisy	ACC_true	Recall	Precision	F1
*	0.3551	0.3823	0.3005	0.7204	0.4230
*+ MGC	0.3781	0.4120	0.3641	0.8306	0.5050
*+ SCAD	0.3797	0.4136	0.3688	0.8286	0.5091
*+ MGC + SCAD	0.3794	0.4133	0.3671	0.8347	0.5087
*+ MCP	0.3784	0.4126	0.3707	0.8255	0.5103
*+ MGC + MCP	0.3791	0.4130	0.3668	0.8327	0.5080
*+ Lasso	0.3778	0.4118	0.3640	0.8296	0.5047
*+ MGC + Lasso	0.3790	0.4130	0.3647	0.8367	0.5067

* represents the *γ*-logistic model, the training data size is 500*1000, and the test data size is 200*1000.

**Table 2:** Results of evaulate metrics on the test data and 0.2 noisy ratio.

	ACC_noisy	ACC_true	Recall	Precision	F1
*	0.3262	0.3826	0.4884	0.7209	0.5807
*+ MGC	0.3420	0.4111	0.5583	0.8245	0.6643
*+ SCAD	0.3443	0.4135	0.5651	0.8270	0.6698
*+ MGC + SCAD	0.3439	0.4130	0.5632	0.8311	0.6699
*+ MCP	0.3434	0.4129	0.5670	0.8311	0.6701
*+ MGC + MCP	0.3440	0.4131	0.5629	0.8306	0.6694
*+ Lasso	0.3426	0.4119	0.5595	0.8296	0.6668
*+ MGC + Lasso	0.3432	0.4124	0.5602	0.8321	0.6680

* represents the *γ*-logistic model, the training data size is 500*1000, and the test data size is 200*1000.

**Table 3:** Results of evaulate metrics on the test data and 0.3 noisy ratio.

	ACC_noisy	ACC_true	Recall	Precision	F1
*	0.2990	0.3824	0.6194	0.7146	0.6623
*+ MGC	0.3070	0.4113	0.6833	0.8218	0.7451
*+ SCAD	0.3088	0.4135	0.6885	0.8262	0.7500
*+ MGC + SCAD	0.3085	0.4129	0.6864	0.8289	0.7499
*+ MCP	0.3079	0.4127	0.6898	0.8224	0.7492
*+ MGC + MCP	0.3085	0.4132	0.6862	0.8279	0.7493
*+ Lasso	0.3073	0.4118	0.6832	0.8259	0.7468
*+ MGC + Lasso	0.3085	0.4131	0.6847	0.8313	0.7498

* represents the *γ*-logistic model, the training data size is 500*1000, and the test data size is 200*1000.

**Table 4:** Results of evaulate metrics on the test data and 0.1 noisy ratio.

	ACC_noisy	ACC_true	Recall	Precision	F1
**	0.3552	0.3824	0.3005	0.7204	0.4230
**+ MGC	0.3779	0.4120	0.3644	0.8316	0.5055
**+ SCAD	0.3798	0.4136	0.3690	0.8296	0.5095
**+ MGC + SCAD	0.3795	0.4134	0.3671	0.8347	0.5087
**+ MCP	0.3783	0.4123	0.3704	0.8255	0.5099
**+ MGC + MCP	0.3792	0.4131	0.3672	0.8337	0.5086
**+ Lasso	0.3778	0.4118	0.3639	0.8296	0.5047
**+ MGC + Lasso	0.3790	0.4130	0.3647	0.8367	0.5067

** represents the probit model, the training data size is 500*1000, and the test data size is 200*1000.

**Table 5:** Results of evaulate metrics on the test data and 0.2 noisy ratio.

	ACC_noisy	ACC_true	Recall	Precision	F1
**+	0.3262	0.3826	0.4882	0.7214	0.5807
**+ MGC	0.3421	0.4111	0.5585	0.8245	0.6644
**+ SCAD	0.3444	0.4136	0.5651	0.8270	0.6698
**+ MGC + SCAD	0.3439	0.4132	0.5632	0.8306	0.6697
**+ MCP	0.3433	0.4126	0.5668	0.8240	0.6699
**+ MGC + MCP	0.3441	0.4132	0.5629	0.8306	0.6694
**+ Lasso	0.3426	0.4120	0.5594	0.8296	0.6667
**+ MGC + Lasso	0.3432	0.4124	0.5602	0.8321	0.6680

** represents the probit model, the training data size is 500*1000, and the test data size is 200*1000.

**Table 6:** Results of evaulate metrics on the test data and 0.3 noisy ratio.

	ACC_noisy	ACC_true	Recall	Precision	F1
**+	0.2990	0.3824	0.6193	0.7153	0.6625
**+ MGC	0.3076	0.4117	0.6830	0.8218	0.7450
**+ SCAD	0.3089	0.4136	0.6885	0.8262	0.7499
**+ MGC + SCAD	0.3085	0.4128	0.6864	0.8289	0.7499
**+ MCP	0.3080	0.4126	0.6896	0.8224	0.7491
**+ MGC + MCP	0.3084	0.4131	0.6857	0.8279	0.7490
**+ Lasso	0.3073	0.4119	0.6829	0.8259	0.7466
**+ MGC + Lasso	0.3085	0.4131	0.6849	0.8313	0.7499

** represents the probit model, the training data size is 500*1000, and the test data size is 200*1000.

**Table 7:** Results of evaluate metrics on the test data and 0.1 noisy ratio.

	ACC_noisy	ACC_true	Recall	Precision	F1
*	0.3612	0.3886	0.3261	0.7061	0.4425
*+ MGC	0.3778	0.4086	0.3577	0.7776	0.4870
*+ SCAD	0.3814	0.4133	0.3688	0.7979	0.5013
*+ MGC + SCAD	0.3835	0.4163	0.3656	0.8041	0.4996
*+ MCP	0.3818	0.4141	0.3680	0.7857	0.4977
*+ MGC + MCP	0.3835	0.4163	0.3665	0.8000	0.4995
*+ Lasso	0.3820	0.4143	0.3644	0.7939	0.4962
*+ MGC + Lasso	0.3833	0.4159	0.3655	0.8041	0.4994

* represents the logistic model, the training data size is 200*500, and the test data size is 100*500.

**Table 8:** Results of evaluate metrics on the test data and 0.2 noisy ratio.

	ACC_noisy	ACC_true	Recall	Precision	F1
*	0.3345	0.3886	0.5208	0.7020	0.5947
*+ MGC	0.3484	0.4100	0.5600	0.7735	0.6464
*+ SCAD	0.3500	0.4139	0.5678	0.7888	0.6573
*+ MGC + SCAD	0.3504	0.4161	0.5646	0.8000	0.6595
*+ MCP	0.3498	0.4141	0.5673	0.7806	0.6538
*+ MGC + MCP	0.3504	0.4157	0.5653	0.7990	0.6594
*+ Lasso	0.3502	0.4141	0.5607	0.7889	0.6527
*+ MGC + Lasso	0.3508	0.4159	0.5621	0.7990	0.6574

* represents the logistic model, the training data size is 200*500, and the test data size is 100*500.

**Table 9:** Results of evaluate metrics on the test data and 0.3 noisy ratio.

	ACC_noisy	ACC_true	Recall	Precision	F1
*	0.3076	0.3886	0.6521	0.6946	0.6700
*+ MGC	0.3182	0.4078	0.6797	0.7639	0.7172
*+ SCAD	0.3189	0.4137	0.6863	0.7776	0.7268
*+ MGC + SCAD	0.3202	0.4171	0.6851	0.7876	0.7306
*+ MCP	0.3188	0.4139	0.6889	0.7707	0.7252
*+ MGC + MCP	0.3194	0.4161	0.6869	0.7864	0.7312
*+ Lasso	0.3192	0.4139	0.6836	0.7809	0.7270
*+ MGC + Lasso	0.3196	0.4155	0.6848	0.7864	0.7301

* represents the logistic model, the training data size is 200*500, and the test data size is 100*500.

**Table 10:** Results of evaluate metrics on the test data and 0.1 noisy ratio.

	ACC_noisy	ACC_true	Recall	Precision	F1
**	0.3547	0.3814	0.3167	0.6878	0.4309
**+ MGC	0.3700	0.4012	0.3539	0.7429	0.4758
**+ SCAD	0.3724	0.4041	0.3597	0.7531	0.4829
**+ MGC + SCAD	0.3741	0.4067	0.3617	0.7694	0.4888
**+ MCP	0.3724	0.4043	0.3584	0.7449	0.4801
**+ MGC + MCP	0.3741	0.4067	0.3620	0.7653	0.4880
**+ Lasso	0.3727	0.4039	0.3552	0.7510	0.4785
**+ MGC + Lasso	0.3749	0.4073	0.3586	0.7714	0.4862

** represents the probit model, the training data size is 200*500, and the test data size is 100*500.

**Table 11:** Results of evaluate metrics on the test data and 0.2 noisy ratio.

	ACC_noisy	ACC_true	Recall	Precision	F1
**	0.3345	0.3886	0.5200	0.7020	0.5941
**+ MGC	0.3480	0.4098	0.5593	0.7755	0.6467
**+ SCAD	0.3496	0.4135	0.5652	0.7898	0.6565
**+ MGC + SCAD	0.3504	0.4163	0.5643	0.8000	0.6592
**+ MCP	0.3498	0.4141	0.5673	0.7806	0.6538
**+ MGC + MCP	0.3504	0.4157	0.5653	0.7990	0.6594
**+ Lasso	0.3502	0.4141	0.5603	0.7888	0.6524
**+ MGC + Lasso	0.3508	0.4159	0.5625	0.8000	0.6580

** represents the probit model, the training data size is 200*500, and the test data size is 100*500.

**Table 12:** Results of evaluate metrics on the test data and 0.3 noisy ratio.

	ACC_noisy	ACC_true	Recall	Precision	F1
**	0.3076	0.3886	0.6514	0.6952	0.6699
**+ MGC	0.3178	0.4080	0.6795	0.7639	0.7172
**+ SCAD	0.3190	0.4135	0.6859	0.7782	0.7270
**+ MGC + SCAD	0.3202	0.4169	0.6856	0.7878	0.7309
**+ MCP	0.3188	0.4139	0.6884	0.7707	0.7249
**+ MGC + MCP	0.3196	0.4161	0.6869	0.7864	0.7312
**+ Lasso	0.3192	0.4139	0.6836	0.7809	0.7270
**+ MGC + Lasso	0.3196	0.4157	0.6843	0.7857	0.7295

** represents the probit model, the training data size is 200*500, and the test data size is 100*500.

**Table 13:** Results of evaluate metrics on the test data and 0.1 noisy ratio.

	ACC_noisy	ACC_true	Recall	Precision	F1
*	0.3552	0.3802	0.2914	0.7388	0.4172
*+ MGC	0.3692	0.3984	0.3474	0.7980	0.4826
*+ SCAD	0.3862	0.4169	0.3679	0.8296	0.5084
*+ MGC + SCAD	\	\	\	\	\
*+ MCP	0.3855	0.4165	0.3695	0.8337	0.5107
*+ MGC + MCP	\	\	\	\	\
*+ Lasso	0.3615	0.3906	0.3417	0.7857	0.4746
*+ MGC + Lasso	0.3599	0.3944	0.3495	0.7937	0.4853

* represents the logistic model, the training data size is 1000*1500, and the test data size is 200*1500. The appearance of the *n* symbol is due to the overflow of operations such as exponentiation, which results in the inability to obtain correct results.

**Table 14:** Results of evaulate metrics on the test data and 0.2 noisy ratio.

	ACC_noisy	ACC_true	Recall	Precision	F1
*	0.3288	0.3804	0.4785	0.7250	0.5755
*+ MGC	0.3295	0.3894	0.5222	0.7709	0.6215
*+ SCAD	0.3526	0.4167	0.5637	0.8219	0.6676
*+ MGC + SCAD	0.3517	0.4158	0.5632	0.8270	0.6690
*+ MCP	0.3521	0.4164	0.5652	0.8245	0.6696
*+ MGC + MCP	\	\	\	\	\
*+ Lasso	0.3298	0.3906	0.5239	0.7755	0.6242
*+ MGC + Lasso	\	\	\	\	\

* represents the logistic model, the training data size is 1000*1500, and the test data size is 200*1500. The appearance of the *n* symbol is due to the overflow of operations such as exponentiation, which results in the inability to obtain correct results.

**Table 15:** Results of evaluate metrics on the test data and 0.3 noisy ratio.

	ACC_noisy	ACC_true	Recall	Precision	F1
*	0.3018	0.3804	0.6123	0.7224	0.6619
*+ MGC	0.3103	0.4064	0.6698	0.8065	0.7308
*+ SCAD	0.3164	0.4165	0.6875	0.8259	0.7493
*+ MGC + SCAD	0.3161	0.4163	0.6870	0.8306	0.7509
*+ MCP	0.3163	0.4164	0.6890	0.8265	0.7505
*+ MGC + MCP	0.3157	0.4157	0.6875	0.8289	0.7505
*+ Lasso	0.2969	0.3907	0.6411	0.7782	0.7019
*+ MGC + Lasso	\	\	\	\	\

* represents the logistic model, the training data size is 1000*1500, and the test data size is 200*1500. The appearance of the *n* symbol is due to the overflow of operations such as exponentiation, which results in the inability to obtain correct results.

**Table 16:** Results of evaluate metrics on the test data and 0.1 noisy ratio.

	ACC_noisy	ACC_true	Recall	Precision	F1
**	0.3552	0.3802	0.2909	0.7388	0.4168
**+ MGC	0.3692	0.3984	0.3475	0.7990	0.4829
**+ SCAD	0.3862	0.4169	0.3679	0.8296	0.5084
**+ MGC + SCAD	0.3854	0.4160	0.3655	0.8327	0.5066
**+ MCP	0.3855	0.4165	0.3695	0.8337	0.5107
**+ MGC + MCP	0.3890	0.4182	0.3774	0.8361	0.5201
**+ Lasso	0.3614	0.3905	0.3419	0.7857	0.4749
**+ MGC + Lasso	\	\	\	\	\

** represents the probit model, the training data size is 1000*1500, and the test data size is 200*1500. The appearance of the *n* symbol is due to the overflow of operations such as exponentiation, which results in the inability to obtain correct results.

**Table 17:** Results of evaulate metrics on the test data and 0.2 noisy ratio.

	ACC_noisy	ACC_true	Recall	Precision	F1
**	0.3288	0.3804	0.4781	0.7255	0.5754
**+ MGC	0.3295	0.3894	0.5223	0.7714	0.6217
**+ SCAD	0.3526	0.4167	0.5637	0.8219	0.6676
**+ MGC + SCAD	0.3518	0.4159	0.5630	0.8270	0.6689
**+ MCP	0.3521	0.4164	0.5652	0.8245	0.6696
**+ MGC + MCP	\	\	\	\	\
**+ Lasso	0.3299	0.3906	0.5240	0.7750	0.6241
**+ MGC + Lasso	\	\	\	\	\

** represents the probit model, the training data size is 1000*1500, and the test data size is 200*1500.

**Table 18:** Results of evaluate metrics on the test data and 0.3 noisy ratio.

	ACC_noisy	ACC_true	Recall	Precision	F1
**	0.3018	0.3804	0.6120	0.7224	0.6617
**+ MGC	0.3104	0.4065	0.6698	0.8068	0.7308
**+ SCAD	0.3165	0.4167	0.6874	0.8255	0.7491
**+ MGC + SCAD	0.3162	0.4163	0.6869	0.8310	0.7510
**+ MCP	0.3163	0.4164	0.6889	0.8265	0.7505
**+ MGC + MCP	0.3155	0.4155	0.6875	0.8289	0.7505
**+ Lasso	0.2970	0.3907	0.6414	0.7786	0.7022
**+ MGC + Lasso	\	\	\	\	\

** represents the probit model, the training data size is 1000*1500, and the test data size is 200*1500.

In addition, by incorporating the proposed meta gradient correction algorithm, the model accuracy on true labels (*ACC*_*true*) and *Precision* are further improved under various simulated scenarios. This demonstrates the effectiveness of the meta gradient correction algorithm in enhancing model robustness and resilience to noisy labels. According to our theoretical analyses (refer to Theorem 4), our model is effective when the noisy ratio is small, which is also validated by the experimental results.

*The analysis of the impact of sample size based on simulated data.* We also evaluated the impact of sample size on model performance. Based on Tables 1–18, we found that when the sample size of the training set increases from 200 to 1000, the predictive ability of the model first increases and then decreases. The reason why the prediction ability improves first is because as the size of the training set increases, the training set contains more practical information. The model with the meta-gradient correction algorithm or the penalty function can capture more information about the correctly labeled samples. As the size of the training set further increases, it is challenging to fit large-scale data sets with simple logistic regression and probability models as the backbone, and they usually require more help to fit such data effectively. At this point, combining the meta-gradient correction algorithm with a penalty model does not provide additional benefits. One potential reason is the inherent conflict between these two technologies’ goals and working mechanisms. When the sample size is small, the excellent capabilities of the two can offset the problems caused by conflicts. When the sample size continues to increase, this problem gradually comes to light. A similar phenomenon occurs when the proportion of noisy labels increases.

*The analysis of the impact of noisy label ratio based on simulated data.* As illustrated in Tables 1–18 and Figs 1–6, the *ACC*_*true* and *Precision* fluctuate severely as the noise ratio increases, especially when the noise level is high (e.g., ≥0.4). In Fig 1, the evaluation metrics show a smooth downward trend when no penalty function exists in the model. In Figs 2–4, the evaluation metrics show a fluctuating downward trend when the model includes the penalty function. These fluctuations directly result from too many noisy labels in the dataset interfering with the features selected by the penalty function, which can cause significant challenges in the training process of models with penalty functions. Therefore, in this case, the model may need help to achieve optimal predictive performance or even converge. In future research efforts, we are actively considering strategies to mitigate these challenges, including introducing smoothing factors or improving penalty functions to enhance model training when dealing with large-scale noisy labels. Further, Figs 1, 2, 5 and 6 show the results of the evaluation metrics for the same model settings and noise labeling ratio settings on the training set and test set, respectively. We find that the performance of the two evaluation metrics, *ACC*_*true* and *Precision*, are roughly the same for the same scenarios, with the performance of the training set being relatively smoother.

In summary, the comprehensive experiments on simulated data validate the effectiveness of our proposed approach in modeling noisy labels and identifying noisy instances. The results are well aligned with our theoretical analyses.

#### Analysis based on real data

*Overall analysis based on real data.* We further benchmark our model on real-world datasets with synthetic noisy labels. As shown in Tables 19–24, the meta gradient correction algorithm consistently improves the results across different noisy ratios and evaluation metrics compared to baseline models. However, the incorporation of penalized models leads to inferior performance. A potential reason is that the real datasets have lower dimensionality and complexity compared to simulated data and, thus, are less prone to overfitting. Therefore, the benefits of regularization models are diminished. Encouragingly, the results further verify the robustness and effectiveness of the proposed meta gradient correction algorithm on real-world noisy label learning tasks.

**Table 19:** Results of evaulate metrics on the breast cancer test data and 0.1 noisy ratio.

	ACC_noisy	ACC_true	Recall	Precision	F1
*	0.0841	0.0541	0.0955	0.9889	0.1742
*+ MGC	0.5166	0.5702	0.1332	0.9999	0.2337
*+ SCAD	0.0747	0.0428	0.0666	0.6698	0.1212
*+ MGC + SCAD	0.5107	0.5630	0.0965	0.9999	0.1760
*+ MCP	0.0746	0.0428	0.0741	0.7514	0.1350
*+ MGC + MCP	0.5113	0.5632	0.0964	0.9999	0.1759
*+ Lasso	0.0752	0.0433	0.0661	0.6642	0.1202
*+ MGC + Lasso	0.5107	0.5630	0.0966	0.9999	0.1761

* represents the *γ*-logistic model, the training data size is 456*31, and the test data size is 115*31.

**Table 20:** Results of evaulate metrics on the breast cancer test data and 0.3 noisy ratio.

	ACC_noisy	ACC_true	Recall	Precision	F1
*	0.1448	0.0547	0.2944	0.9819	0.4531
*+ MGC	0.4153	0.5814	0.3774	0.9999	0.5452
*+ SCAD	0.1399	0.0429	0.2279	0.6963	0.3434
*+ MGC + SCAD	0.3983	0.5464	0.2982	0.9999	0.4594
*+ MCP	0.1396	0.0427	0.2454	0.7665	0.3717
*+ MGC + MCP	0.3901	0.5372	0.2981	0.9999	0.4593
*+ Lasso	0.1402	0.0435	0.2254	0.6867	0.3394
*+ MGC + Lasso	0.3984	0.5463	0.2983	0.9999	0.4596

* represents the *γ*-logistic model, the training data size is 456*31, and the test data size is 115*31.

**Table 21:** Results of evaulate metrics on the breast cancer test data and 0.5 noisy ratio.

	ACC_noisy	ACC_true	Recall	Precision	F1
*	0.2049	0.0551	0.4951	0.9810	0.6580
*+ MGC	0.3004	0.5818	0.5636	0.9999	0.7196
*+ SCAD	0.2027	0.0431	0.4093	0.6953	0.5151
*+ MGC + SCAD	0.2975	0.5736	0.4999	0.9999	0.6665
*+ MCP	0.2022	0.0427	0.4314	0.7604	0.5503
*+ MGC + MCP	0.2971	0.5751	0.5001	0.9999	0.6667
*+ Lasso	0.2026	0.0437	0.4047	0.6824	0.5079
*+ MGC + Lasso	0.2975	0.5736	0.5000	0.9999	0.6666

* represents the *γ*-logistic model, the training data size is 456*31, and the test data size is 115*31.

**Table 22:** Results of evaulate metrics on the breast cancer test data and 0.1 noisy ratio.

	ACC_noisy	ACC_true	Recall	Precision	F1
**	0.0841	0.0542	0.0956	0.9888	0.1743
**+ MGC	0.5167	0.5701	0.1329	0.9999	0.2333
**+ SCAD	0.0746	0.0427	0.0665	0.6697	0.1211
**+ MGC + SCAD	0.5193	0.5729	0.0966	0.9999	0.1761
**+ MCP	0.0748	0.0429	0.0740	0.7515	0.1351
**+ MGC + MCP	0.5138	0.5673	0.0964	0.9998	0.1758
**+ Lasso	0.0753	0.0434	0.0660	0.6641	0.1201
**+ MGC + Lasso	0.5193	0.5729	0.0966	0.9998	0.1759

** represents the probit model, the training data size is 456*31, and the test data size is 115*31.

**Table 23:** Results of evaulate metrics on the breast cancer test data and 0.3 noisy ratio.

	ACC_noisy	ACC_true	Recall	Precision	F1
**	0.1448	0.0548	0.2944	0.9820	0.4530
**+ MGC	0.4153	0.5813	0.3771	0.9999	0.5450
**+ SCAD	0.1400	0.0430	0.2280	0.6969	0.3435
**+ MGC + SCAD	0.3972	0.5478	0.2982	0.9999	0.4595
**+ MCP	0.1396	0.0428	0.2455	0.7671	0.3719
**+ MGC + MCP	0.3946	0.5426	0.2983	0.9998	0.4594
**+ Lasso	0.1402	0.0435	0.2253	0.6861	0.3391
**+ MGC + Lasso	0.3972	0.5478	0.2984	0.9998	0.4596

** represents the probit model, the training data size is 456*31, and the test data size is 115*31.

**Table 24:** Results of evaulate metrics on the breast cancer test data and 0.5 noisy ratio.

	ACC_noisy	ACC_true	Recall	Precision	F1
**	0.2050	0.0551	0.4952	0.9810	0.6581
**+ MGC	0.3004	0.5818	0.5636	0.9999	0.7197
**+ SCAD	0.2026	0.0431	0.4092	0.6950	0.5149
**+ MGC + SCAD	0.2981	0.5723	0.5000	0.9999	0.6667
**+ MCP	0.2023	0.0428	0.4315	0.7605	0.5504
**+ MGC + MCP	0.2986	0.5745	0.5000	0.9998	0.6666
**+ Lasso	0.2026	0.0437	0.4049	0.6828	0.5081
**+ MGC + Lasso	0.2981	0.5723	0.5000	0.9998	0.6666

** represents the probit model, the training data size is 456*31, and the test data size is 115*31.

In conclusion, extensive experiments on both simulated and real-world datasets demonstrate the capability of our model in handling noisy labels. The meta gradient correction algorithm is verified to deliver consistent performance improvements. In future works, we will focus on alleviating the model fluctuation issue when the noise level is high and evaluating the approach on larger-scale and more complex real-world data. Advanced deep learning techniques can also be explored as backbone models to enhance model capacity and scalability.

## Conclusion and future work

In this paper, we have proposed a novel penalized model and a meta gradient correction algorithm with grounded theoretical foundations to detect noisy labels. To illustrate the effectiveness of our proposed model and algorithm, we argue from both theoretical proofs and rich experiments.

First, we derive theorems 1 and 2, which reveal that the $\hat{\beta }$ in the proposed model possesses consistency and asymptotic normality. Besides, we obtain Theorem 3 based on the empirical risk upper bound derivation, proving that the difference between our model and the optimal Bayesian model is sufficiently small. Furthermore, we propose a meta-learning algorithm for correcting gradient and show its convergence based on rigorous theoretical foundations (refer to Theorem 4).

Next, we conducted experiments on multiple simulated and real data. These experiments proved the following results: **(i)** The proposed penalized model exhibits surprising robustness in modeling noisy labels. **(ii)** The proposed meta gradient correction algorithm demonstrates a promising ability to detect and find noisy labels. **(iii)** Our model can easily used in practical machine learning scenarios.

Finally, we plan to improve our algorithm to make it suitable for large-scale high-dimensional data. Possible improvements include introducing more complex models and smoother optimization algorithms. In addition, our algorithm can also be used in other fields, such as casual inference.

## Supporting information

S1 File The title of this file is ‘Supprot information for “Robust meta gradient learning for high-dimensional data with noisy-label ignorance”’. The support information file contains all the proofs covered by the manuscript. Specifically, it contains the derivation of Eq (5) and the proofs of 1, 2, 3, and 4.
(PDF)
