# Supplementary material for: Robust meta gradient learning for high-dimensional data with noisy-label ignorance
Source: PLoS One. 2023 Dec 11;18(12):e0295678. doi: 10.1371/journal.pone.0295678 (PMC10712856; doi:10.1371/journal.pone.0295678)
Supplement: S1 File — The support information file contains all the proofs covered by the manuscript. Specifically, it contains the derivation of Eq (5) and the proofs of 1, 2, 3, and 4. (PDF) [file pone.0295678.s001.pdf]

Supprot information for "Robust meta gradient learning for high-dimensional data with noisy-label ignorance"

Ben Liu[1], Yu Lin[1*]

1 School of Statistics, Southwestern University of Finance and Economics, Chengdu, Sichuan, China

* 2170202j8001@smail.swufe.edu.cn

S1: The Derivation of Eq.(1)

Proof. Recall that

$$g(y|x) = c(x)f(y|x;\boldsymbol{\beta}_0) + (1-c(x))h(y|x), \tag{A.1}$$

and

$$h(y|x) = (\frac{\eta_0(x)}{\eta_0(x)+\eta_1(x)})^y(\frac{\eta_1(x)}{\eta_0(x)+\eta_1(x)})^{1-y}. \tag{A.2}$$

With some calculations, it can be shown that

$$\begin{aligned}
D_\gamma(g(y|x), f(y|x;\boldsymbol{\beta})) &= \frac{1}{\gamma(\gamma+1)}\Big[||g(y|x)||_{\gamma+1} - \int(\frac{f(y|x;\boldsymbol{\beta})}{||f(\cdot|x;\boldsymbol{\beta})||_{\gamma+1}})^\gamma g(y|x)\Big] \\
&= \frac{1}{\gamma(\gamma+1)}\Big[\{\int(c(x)f(y|x;\boldsymbol{\beta}_0)+(1-c(x))h(y|x))^{\gamma+1}\}^{\frac{1}{\gamma+1}} \\
&\quad - \int(\frac{f(y|x;\boldsymbol{\beta})}{||f(\cdot|x;\boldsymbol{\beta})||_{\gamma+1}})^\gamma c(x)f(y|x;\boldsymbol{\beta}_0) \\
&\quad - \int(\frac{f(y|x;\boldsymbol{\beta})}{||f(\cdot|x;\boldsymbol{\beta})||_{\gamma+1}})^\gamma (1-c(x))h(y|x)\Big] \\
&= \frac{1}{\gamma(\gamma+1)}\Big\{\int\big(c(x)f(y|x;\boldsymbol{\beta}_0)+(1-c(x))h(y)\big)^{\gamma+1}\Big\}^{\frac{1}{\gamma+1}} \\
&\quad - \frac{1}{\gamma(\gamma+1)}c(x)||f(y|x;\boldsymbol{\beta}_0)||_{\gamma+1} \\
&\quad + \frac{1}{\gamma(\gamma+1)}c(x)||f(y|x;\boldsymbol{\beta}_0)||_{\gamma+1} \\
&\quad - \frac{1}{\gamma(\gamma+1)}\int\Big(\frac{f(y|x;\boldsymbol{\beta})}{||f(\cdot|x;\boldsymbol{\beta})||_{\gamma+1}}\Big)^\gamma c(x)f(y|x;\boldsymbol{\beta}_0) \\
&\quad - \frac{1}{\gamma(\gamma+1)}\int\Big(\frac{f(y|x;\boldsymbol{\beta})}{||f(\cdot|x;\boldsymbol{\beta})||_{\gamma+1}}\Big)^\gamma (1-c(x))h(y|x) \\
&= \Big[c(x)D_\gamma\{f(y|x;\boldsymbol{\beta}_0), f(y|x;\boldsymbol{\beta})\} - \frac{B_\gamma(c(x),h(y|x),\boldsymbol{\beta})}{\gamma(\gamma+1)}\Big] \\
&\quad + \frac{||c(x)f(y|x;\boldsymbol{\beta}_0)+(1-c(x))h(y)||_{\gamma+1} - c(x)||f(y|x;\boldsymbol{\beta}_0)||_{\gamma+1}}{\gamma(\gamma+1)},
\end{aligned} \tag{A.3}$$

where

$$B_\gamma(c(x),h(y|x),\boldsymbol{\beta}) = (1-c(x))\int(\frac{f(y|x;\boldsymbol{\beta})}{||f(\cdot|x;\boldsymbol{\beta})||_{\gamma+1}})^\gamma h(y|x). \tag{A.4}$$

Because the third term in the above formula has no effect on the estimation of $\boldsymbol{\beta}$, minimizing the above formula over $\boldsymbol{\beta}$ is equivalent to minimizing

$$cD_\gamma(f(y|x;\boldsymbol{\beta}_0), f(y|x;\boldsymbol{\beta})) - \frac{B_\gamma(c(x),h(y|x),\boldsymbol{\beta})}{\gamma(\gamma+1)}. \tag{A.5}$$

Notice that,

$$
\begin{aligned}
B_\gamma(c(x), h(y|x), \boldsymbol{\beta}) &= (1-c(x))\int (\frac{f(y|x;\boldsymbol{\beta})}{||f(\cdot|x;\boldsymbol{\beta})||_{\gamma+1}})^\gamma h(y|x) \\
&= \eta_0(x)(\frac{\pi(x;\boldsymbol{\beta})}{||f(\cdot|x;\boldsymbol{\beta})||_{\gamma+1}})^\gamma + \eta_1(x)(\frac{1-\pi(x;\boldsymbol{\beta})}{||f(\cdot|x;\boldsymbol{\beta})||_{\gamma+1}})^\gamma \\
&= \frac{\eta_0(x)\{\pi(x;\boldsymbol{\beta})\}^\gamma + \eta_1(x)\{1-\pi(x;\boldsymbol{\beta})\}^\gamma}{[\{\pi(x;\boldsymbol{\beta})\}^{\gamma+1} + \{1-\pi(x;\boldsymbol{\beta})\}^{\gamma+1}]^{\frac{\gamma}{\gamma+1}}}.
\end{aligned}
\tag{A.6}
$$

From power mean inequality [1],

$$
\Big[\{\pi(x;\boldsymbol{\beta})\}^{\gamma+1} + \{1-\pi(x;\boldsymbol{\beta})\}^{\gamma+1}\Big]^{\frac{1}{\gamma+1}} \geq \Big[\{\pi(x;\boldsymbol{\beta})\}^{\gamma} + \{1-\pi(x;\boldsymbol{\beta})\}^{\gamma}\Big]^{\frac{1}{\gamma}}. \tag{A.7}
$$

Hence, we have

$$
\begin{aligned}
0 &\leq \frac{B_\gamma(c(x), h(y|x), \boldsymbol{\beta})}{\gamma(\gamma+1)} \\
&\leq \frac{\eta_0(x)\{\pi(x;\boldsymbol{\beta})\}^\gamma + \eta_1(x)\{1-\pi(x;\boldsymbol{\beta})\}^\gamma}{\gamma(\gamma+1)[\{\pi(x;\boldsymbol{\beta})\}^\gamma + \{1-\pi(x;\boldsymbol{\beta})\}^\gamma]} \\
&= \frac{\eta_0(x)}{\gamma(\gamma+1)} + \frac{\eta_1(x)-\eta_0(x)}{\gamma(\gamma+1)(\frac{\pi(x;\boldsymbol{\beta})}{1-\pi(x;\boldsymbol{\beta})})^\gamma + \gamma(\gamma+1)} \to\ 0,
\end{aligned}
\tag{A.8}
$$

as $\gamma \to \infty$. At last, we obtain that

$$
\begin{aligned}
D_\gamma(g(y|x), f(y|x;\boldsymbol{\beta})) &\propto cD_\gamma(f(y|x;\boldsymbol{\beta}_0), f(y|x;\boldsymbol{\beta})) \\
&\quad - \frac{B_\gamma(c(x), h(y|x), \boldsymbol{\beta})}{\gamma(\gamma+1)} \\
&\approx\ cD_\gamma(f(y|x;\boldsymbol{\beta}_0), f(y|x;\boldsymbol{\beta})).
\end{aligned}
\tag{A.9}
$$

By the definition of γ-divergence,

$$
D_\gamma(f(y|x;\boldsymbol{\beta}_0), f(y|x;\boldsymbol{\beta})) = \frac{1}{\gamma(\gamma+1)}\Bigg[||f(y|x;\boldsymbol{\beta}_0)||_{\gamma+1} - \int\Big\{\frac{f(y|x;\boldsymbol{\beta})}{||f(\cdot|x;\boldsymbol{\beta})||_{\gamma+1}}\Big\}^\gamma f(y|x;\boldsymbol{\beta}_0)\Bigg]. \tag{A.10}
$$

So, based on (A.9) and (A.10), we have

$$
\begin{aligned}
\arg\min_{\boldsymbol{\beta}} D_\gamma(g(y|x), f(y|x;\boldsymbol{\beta})) &= \arg\min_{\boldsymbol{\beta}}\ c(x)D_\gamma(f(y|x;\boldsymbol{\beta}_0), f(y|x;\boldsymbol{\beta})) \\
&= \arg\max_{\boldsymbol{\beta}} \int\Big\{\frac{f(y|x;\boldsymbol{\beta})}{||f(\cdot|x;\boldsymbol{\beta})||_{\gamma+1}}\Big\}^\gamma f(y|x;\boldsymbol{\beta}_0).
\end{aligned}
\tag{A.11}
$$

As stated in section of methodology, we take the expectations with respect to X on right side of the above formula to get that

$$
\arg\min_{\boldsymbol{\beta}}\ E_X\big[D_\gamma(g(y|x), f(y|x;\boldsymbol{\beta}))\big] = \arg\max_{\boldsymbol{\beta}} E_{X,Y}\Bigg[\Big\{\frac{f(y|x;\boldsymbol{\beta})}{||f(\cdot|x;\boldsymbol{\beta})||_{\gamma+1}}\Big\}^\gamma\Bigg]. \tag{A.12}
$$

□

S2: Proofs of Lemmas 1 and 2

Proof. First, we get

$$
\begin{aligned}
\frac{\partial \phi(\boldsymbol{\beta})}{\partial \boldsymbol{\beta}} &= \frac{\gamma[\{\pi(X_i;\boldsymbol{\beta})\}^{Y_i}\{1-\pi(X_i;\boldsymbol{\beta})\}^{1-Y_i}]^{\gamma-1}[Y_i\{\pi(X_i;\boldsymbol{\beta})\}^{Y_i-1}}{[\{\pi(X_i;\boldsymbol{\beta})\}^{\gamma+1}+\{1-\pi(X_i;\boldsymbol{\beta})\}^{\gamma+1}]^{\frac{\gamma}{\gamma+1}}} \\
&\cdot \frac{\pi^{'}(X_i;\boldsymbol{\beta})\{1-\pi(X_i;\boldsymbol{\beta})\}^{1-Y_i}]}{[\{\pi(X_i;\boldsymbol{\beta})\}^{\gamma+1}+\{1-\pi(X_i;\boldsymbol{\beta})\}^{\gamma+1}]^{\frac{\gamma}{\gamma+1}}} \\
&- \frac{\gamma[\{\pi(X_i;\boldsymbol{\beta})\}^{Y_i}\{1-\pi(X_i;\boldsymbol{\beta})\}^{1-Y_i}]^{\gamma-1}[(1-Y_i)\{\pi(X_i;\boldsymbol{\beta})\}^{Y_i}}{[\{\pi(X_i;\boldsymbol{\beta})\}^{\gamma+1}+\{1-\pi(X_i;\boldsymbol{\beta})\}^{\gamma+1}]^{\frac{\gamma}{\gamma+1}}} \\
&\cdot \frac{\{1-\pi(X_i;\boldsymbol{\beta})\}^{-Y_i}\pi^{'}(X_i;\boldsymbol{\beta})]}{[\{\pi(X_i;\boldsymbol{\beta})\}^{\gamma+1}+\{1-\pi(X_i;\boldsymbol{\beta})\}^{\gamma+1}]^{\frac{\gamma}{\gamma+1}}} \\
&- \Big[\frac{\gamma}{\gamma+1}\frac{[\{\pi(X_i;\boldsymbol{\beta})\}^{Y_i}\{1-\pi(X_i;\boldsymbol{\beta})\}^{1-Y_i}]^{\gamma}}{[\{\pi(X_i;\boldsymbol{\beta})\}^{\gamma+1}+\{1-\pi(X_i;\boldsymbol{\beta})\}^{\gamma+1}]^{\frac{2\gamma+1}{\gamma+1}}} \\
&\cdot [(\gamma+1)\{\pi(X_i;\boldsymbol{\beta})\}^{\gamma}\pi^{'}(X_i;\boldsymbol{\beta})]\Big] \\
&+ \Big[\frac{\gamma}{\gamma+1}\frac{[\{\pi(X_i;\boldsymbol{\beta})\}^{Y_i}\{1-\pi(X_i;\boldsymbol{\beta})\}^{1-Y_i}]^{\gamma}}{[\{\pi(X_i;\boldsymbol{\beta})\}^{\gamma+1}+\{1-\pi(X_i;\boldsymbol{\beta})\}^{\gamma+1}]^{\frac{2\gamma+1}{\gamma+1}}} \\
&\cdot [(\gamma+1)\{1-\pi(X_i;\boldsymbol{\beta})\}^{\gamma}\pi^{'}(X_i;\boldsymbol{\beta})]\Big] \\
&= \frac{\gamma Y_i\{\pi(X_i;\boldsymbol{\beta})\}^{Y_i\gamma+\gamma}\{1-\pi(X_i;\boldsymbol{\beta})\}^{\gamma-\gamma Y_i}\pi^{'}(X_i;\boldsymbol{\beta})}{[\{\pi(X_i;\boldsymbol{\beta})\}^{\gamma+1}+\{1-\pi(X_i;\boldsymbol{\beta})\}^{\gamma+1}]^{\frac{2\gamma+1}{\gamma+1}}} \\
&- \frac{\gamma(1-Y_i)\{\pi(X_i;\boldsymbol{\beta})\}^{Y_i\gamma+\gamma+1}\{1-\pi(X_i;\boldsymbol{\beta})\}^{\gamma-\gamma Y_i-1}\pi^{'}(X_i;\boldsymbol{\beta})}{[\{\pi(X_i;\boldsymbol{\beta})\}^{\gamma+1}+\{1-\pi(X_i;\boldsymbol{\beta})\}^{\gamma+1}]^{\frac{2\gamma+1}{\gamma+1}}} \\
&+ \frac{\gamma Y_i\{\pi(X_i;\boldsymbol{\beta})\}^{Y_i\gamma-1}\{1-\pi(X_i;\boldsymbol{\beta})\}^{2\gamma-\gamma Y_i+1}\pi^{'}(X_i;\boldsymbol{\beta})}{[\{\pi(X_i;\boldsymbol{\beta})\}^{\gamma+1}+\{1-\pi(X_i;\boldsymbol{\beta})\}^{\gamma+1}]^{\frac{2\gamma+1}{\gamma+1}}} \\
&- \frac{\gamma(1-Y_i)\{\pi(X_i;\boldsymbol{\beta})\}^{Y_i\gamma}\{1-\pi(X_i;\boldsymbol{\beta})\}^{2\gamma-\gamma Y_i}\pi^{'}(X_i;\boldsymbol{\beta})}{[\{\pi(X_i;\boldsymbol{\beta})\}^{\gamma+1}+\{1-\pi(X_i;\boldsymbol{\beta})\}^{\gamma+1}]^{\frac{2\gamma+1}{\gamma+1}}} \\
&- \frac{\gamma\{\pi(X_i;\boldsymbol{\beta})\}^{Y_i\gamma+\gamma}\{1-\pi(X_i;\boldsymbol{\beta})\}^{\gamma-\gamma Y_i}\pi^{'}(X_i;\boldsymbol{\beta})}{[\{\pi(X_i;\boldsymbol{\beta})\}^{\gamma+1}+\{1-\pi(X_i;\boldsymbol{\beta})\}^{\gamma+1}]^{\frac{2\gamma+1}{\gamma+1}}} \\
&+ \frac{\gamma\{\pi(X_i;\boldsymbol{\beta})\}^{Y_i\gamma}\{1-\pi(X_i;\boldsymbol{\beta})\}^{2\gamma-\gamma Y_i}\pi^{'}(X_i;\boldsymbol{\beta})}{[\{\pi(X_i;\boldsymbol{\beta})\}^{\gamma+1}+\{1-\pi(X_i;\boldsymbol{\beta})\}^{\gamma+1}]^{\frac{2\gamma+1}{\gamma+1}}} \\
&= \frac{\gamma\pi^{'}(X_i;\boldsymbol{\beta})Y_i\{\pi(X_i;\boldsymbol{\beta})\}^{Y_i\gamma+\gamma}\{1-\pi(X_i;\boldsymbol{\beta})\}^{\gamma-\gamma Y_i}}{[\{\pi(X_i;\boldsymbol{\beta})\}^{\gamma+1}+\{1-\pi(X_i;\boldsymbol{\beta})\}^{\gamma+1}]^{\frac{2\gamma+1}{\gamma+1}}} \\
&+ \frac{\gamma\pi^{'}(X_i;\boldsymbol{\beta})Y_i\{\pi(X_i;\boldsymbol{\beta})\}^{Y_i\gamma-1}\{1-\pi(X_i;\boldsymbol{\beta})\}^{2\gamma-\gamma Y_i-1}}{[\{\pi(X_i;\boldsymbol{\beta})\}^{\gamma+1}+\{1-\pi(X_i;\boldsymbol{\beta})\}^{\gamma+1}]^{\frac{2\gamma+1}{\gamma+1}}} \\
&- \frac{\gamma\pi^{'}(X_i;\boldsymbol{\beta})[\{\pi(X_i;\boldsymbol{\beta})\}^{Y_i\gamma+\gamma}\{1-\pi(X_i;\boldsymbol{\beta})\}^{\gamma-\gamma Y_i}]}{[\{\pi(X_i;\boldsymbol{\beta})\}^{\gamma+1}+\{1-\pi(X_i;\boldsymbol{\beta})\}^{\gamma+1}]^{\frac{2\gamma+1}{\gamma+1}}} \\
&- \frac{\gamma\pi^{'}(X_i;\boldsymbol{\beta})[\{\pi(X_i;\boldsymbol{\beta})\}^{Y_i\gamma+\gamma+1}\{1-\pi(X_i;\boldsymbol{\beta})\}^{\gamma-\gamma Y_i-1}(1-Y_i)]}{[\{\pi(X_i;\boldsymbol{\beta})\}^{\gamma+1}+\{1-\pi(X_i;\boldsymbol{\beta})\}^{\gamma+1}]^{\frac{2\gamma+1}{\gamma+1}}} \\
&- \frac{\gamma\pi^{'}(X_i;\boldsymbol{\beta})[(1-Y_i)\{\pi(X_i;\boldsymbol{\beta})\}^{Y_i\gamma}\{1-\pi(X_i;\boldsymbol{\beta})\}^{2\gamma-\gamma Y_i}]}{[\{\pi(X_i;\boldsymbol{\beta})\}^{\gamma+1}+\{1-\pi(X_i;\boldsymbol{\beta})\}^{\gamma+1}]^{\frac{2\gamma+1}{\gamma+1}}} \\
&+ \frac{\gamma\pi^{'}(X_i;\boldsymbol{\beta})[\{\pi(X_i;\boldsymbol{\beta})\}^{Y_i\gamma}\{1-\pi(X_i;\boldsymbol{\beta})\}^{2\gamma-\gamma Y_i}]}{[\{\pi(X_i;\boldsymbol{\beta})\}^{\gamma+1}+\{1-\pi(X_i;\boldsymbol{\beta})\}^{\gamma+1}]^{\frac{2\gamma+1}{\gamma+1}}}
\end{aligned}
$$

$$= \frac{\gamma\pi^{'}(X_i;\boldsymbol{\beta})[\{\pi(X_i;\boldsymbol{\beta})\}^{Y_i\gamma+\gamma}\{1-\pi(X_i;\boldsymbol{\beta})\}^{\gamma-\gamma Y_i}(Y_i+Y_i(\frac{1-\pi(X_i;\boldsymbol{\beta})}{\pi(X_i;\boldsymbol{\beta})})^{\gamma+1}-1)]}{[\{\pi(X_i;\boldsymbol{\beta})\}^{\gamma+1}+\{1-\pi(X_i;\boldsymbol{\beta})\}^{\gamma+1}]^{\frac{2\gamma+1}{\gamma+1}}} - \frac{\gamma\pi^{'}(X_i;\boldsymbol{\beta})[\{\pi(X_i;\boldsymbol{\beta})\}^{Y_i\gamma}\{1-\pi(X_i;\boldsymbol{\beta})\}^{2\gamma-\gamma Y_i}((1-Y_i)(\frac{\pi(X_i;\boldsymbol{\beta})}{1-\pi(X_i;\boldsymbol{\beta})})^{\gamma+1}-Y_i)]}{[\{\pi(X_i;\boldsymbol{\beta})\}^{\gamma+1}+\{1-\pi(X_i;\boldsymbol{\beta})\}^{\gamma+1}]^{\frac{2\gamma+1}{\gamma+1}}}. \tag{B.1}$$

To simplify the calculation, we let $z=\pi(X_i;\boldsymbol{\beta})$ and the above formula is equivalent to

$$\frac{\partial\phi(\boldsymbol{\beta})}{\partial\boldsymbol{\beta}} = \frac{\gamma z^{'}[z^{Y_i\gamma+\gamma}(1-z)^{\gamma-\gamma Y_i}(Y_i+Y_i(\frac{(1-z)}{z})^{\gamma+1}-1)]}{[z^{\gamma+1}+(1-z)^{\gamma+1}]^{\frac{2\gamma+1}{\gamma+1}}} - \frac{\gamma z^{'}[z^{Y_i\gamma}(1-z)^{2\gamma-\gamma Y_i}((1-Y_i)(\frac{z}{(1-z)})^{\gamma+1}-Y_i)]}{[z^{\gamma+1}+(1-z)^{\gamma+1}]^{\frac{2\gamma+1}{\gamma+1}}}. \tag{B.2}$$

And then take the expectation, we get

$$\begin{aligned} E\Big(\frac{\partial\phi(\boldsymbol{\beta})}{\partial\boldsymbol{\beta}}\Big) &= \frac{\gamma z^{'}E\Big[z^{Y_i\gamma+\gamma}(1-z)^{\gamma-\gamma Y_i}Y_i+z^{Y_i\gamma+\gamma}(1-z)^{\gamma-\gamma Y_i}Y_i(\frac{(1-z)}{z})^{\gamma+1}-z^{Y_i\gamma+\gamma}(1-z)^{\gamma-\gamma Y_i}\Big]}{[z^{\gamma+1}+(1-z)^{\gamma+1}]^{\frac{2\gamma+1}{\gamma+1}}} \\ &\quad - \frac{\gamma z^{'}E\Big[z^{Y_i\gamma}(1-z)^{2\gamma-\gamma Y_i}(1-Y_i)(\frac{z}{(1-z)})^{\gamma+1}-z^{Y_i\gamma}(1-z)^{2\gamma-\gamma Y_i}Y_i\Big]}{[z^{\gamma+1}+(1-z)^{\gamma+1}]^{\frac{2\gamma+1}{\gamma+1}}} \\ &= \frac{\gamma z^{'}\Big[z^{2\gamma}P(Y_i=1)+z^{2\gamma}(\frac{1-z}{z})^{\gamma+1}P(Y_i=1)-z^{2\gamma}P(Y_i=1)-z^{\gamma}(1-z)^{\gamma}P(Y_i=0)\Big]}{[z^{\gamma+1}+(1-z)^{\gamma+1}]^{\frac{2\gamma+1}{\gamma+1}}} \\ &\quad - \frac{\gamma z^{'}\Big[(1-z)^{2\gamma}(\frac{z}{1-z})^{\gamma+1}P(Y_i=0)-z^{\gamma}(1-z)^{\gamma}P(Y_i)=1\Big]}{[z^{\gamma+1}+(1-z)^{\gamma+1}]^{\frac{2\gamma+1}{\gamma+1}}} \\ &= \frac{\gamma z^{'}}{[z^{\gamma+1}+(1-z)^{\gamma+1}]^{\frac{2\gamma+1}{\gamma+1}}}\Big[z^{\gamma-1}(1-z)^{\gamma+1}P(Y_i=1)-z^{\gamma}(1-z)^{\gamma}P(Y_i=0) \\ &\quad -(1-z)^{\gamma-1}z^{\gamma+1}P(Y_i=0)+z^{\gamma}(1-z)^{\gamma}P(Y_i=1)\Big]. \end{aligned} \tag{B.3}$$

Moreover, we substitute $P(Y_i=1)=\pi(X_i;\boldsymbol{\beta}_0)$ and $\boldsymbol{\beta}=\boldsymbol{\beta}_0$ into the above formula and get $E\Big(\frac{\partial\phi(\boldsymbol{\beta}_0)}{\partial\boldsymbol{\beta}}\Big)=0$. This completes the proof of Lemma 1.

Next, by taking the first derivative, we have

$$\begin{aligned} E\Big[\{\frac{\partial\phi(\boldsymbol{\beta})}{\partial\boldsymbol{\beta}}\}\{\frac{\partial\phi(\boldsymbol{\beta})}{\partial\boldsymbol{\beta}}\}^{\mathrm{T}}\Big] &= \frac{\gamma^2z^{2\gamma}(1-z)^{2\gamma}\frac{1}{(1-z)^2z^2}z^{'}E\Big[(\frac{z}{1-z})^{2Y_i\gamma}((z^{\gamma+1}+(1-z)^{\gamma+1})Y_i-z^{\gamma+1})^2\Big](z^{'})^{\mathrm{T}}}{[z^{\gamma+1}+(1-z)^{\gamma+1}]^{\frac{4\gamma+2}{\gamma+1}}} \\ &= \frac{\gamma^2z^{2\gamma}(1-z)^{2\gamma}\frac{1}{(1-z)^2z^2}z^{'}\Big[(1-z)^{2\gamma+2}zP(Y_i=1)+z^{2\gamma+2}P(Y_i=0)\Big](z^{'})^{\mathrm{T}}}{[z^{\gamma+1}+(1-z)^{\gamma+1}]^{\frac{4\gamma+2}{\gamma+1}}}, \end{aligned} \tag{B.4}$$

where $z=\pi(X_i;\boldsymbol{\beta})$. Because $(1-z)^{2\gamma+2}zP(Y_i=1)+z^{2\gamma+2}P(Y_i=0)>0$ always holds, then $I(\boldsymbol{\beta})$ is positive definite at $\boldsymbol{\beta}=\boldsymbol{\beta}_0$, this completes the proof of Lemma 2. □

S3: Proofs of Theorems 1 and 2

Proof. Let $\alpha_n=n^{-\frac{1}{2}}+a_n$. For any $\epsilon>0$, if we can show that there exists a local maximum in the ball $\{\boldsymbol{\beta}_0+\alpha_n u:\|u\|\le C\}$ for a sufficiently large constant C with

probability at least $1-\epsilon$, then we have $\|\hat{\boldsymbol{\beta}}-\boldsymbol{\beta}_0\| = O_{\mathrm{p}}(\alpha_n)$. Thus, it suffices to show that

$$P\Big\{\sup_{\|u\|=C} Q(\boldsymbol{\beta}_0+\alpha_n u) < Q(\boldsymbol{\beta}_0)\Big\} \geq 1-\epsilon\,. \tag{C.1}$$

In the sequel, we will show (C.1). Let $D_n(u) = Q(\boldsymbol{\beta}_0+\alpha_n u) - Q(\boldsymbol{\beta}_0)$. It holds that

$$\begin{aligned} D_n(u) &\leq L(\boldsymbol{\beta}_0+\alpha_n u) - L(\boldsymbol{\beta}_0) - n\sum_{j=1}^{s}\Big\{P_{\lambda_n}(|\beta_{j0}+\alpha_n u_j|) - P_{\lambda_n}(|\beta_{j0}|)\Big\} \\ &= \alpha_n L^{'}(\boldsymbol{\beta}_0)^{\mathrm{T}} u + \frac{1}{2}\alpha_n^2 u^{\mathrm{T}} L^{''}(\boldsymbol{\beta}_0) u\{1+o_{\mathrm{p}}(1)\} \\ &\quad - n\sum_{j=1}^{s}\Big[\alpha_n P^{'}_{\lambda_n}(|\beta_{j0}|) sgn(\beta_{j0}) u_j + \frac{1}{2}\alpha_n^2 u_j^2 P^{''}_{\lambda_n}(|\beta_{j0}|)\{1+o(1)\}\Big] \\ &= \alpha_n L^{'}(\boldsymbol{\beta}_0)^{\mathrm{T}} u - \frac{n}{2}\alpha_n^2 u^{\mathrm{T}} I(\boldsymbol{\beta}_0) u\{1+o_{\mathrm{p}}(1)\} \\ &\quad - n\sum_{j=1}^{s}\Big[\alpha_n P^{'}_{\lambda_n}(|\beta_{j0}|) sgn(\beta_{j0}) u_j + \frac{1}{2}\alpha_n^2 u_j^2 P^{''}_{\lambda_n}(|\beta_{j0}|)\{1+o(1)\}\Big], \end{aligned} \tag{C.2}$$

where the first inequality holds by the fact that $P_{\lambda_n}(0)=0$ and $\boldsymbol{\beta}_{20}=0$, and the second equation holds by the Taylor expansion.

By Lemma 1 and 2, we have $n^{-\frac{1}{2}} L^{'}(\boldsymbol{\beta}_0)^{\mathrm{T}} = O_{\mathrm{p}}(1)$. Hence, the order of the first term on the right-hand side of (C.2) is $O_{\mathrm{p}}(n\alpha_n^2)$ provided that $a_n = o_{\mathrm{p}}(n^{-\frac{1}{2}})$. By Lemma 2, $n\alpha_n^2\{1+o_{\mathrm{p}}(1)\}I(\boldsymbol{\beta}_0) = O_{\mathrm{p}}(n\alpha_n^2)$ and $\frac{1}{2}n\alpha_n^2 u^{\mathrm{T}} I(\boldsymbol{\beta}_0)u\{1+o_{\mathrm{p}}(1)\} > 0$, then the second term dominates the first term on the right-hand side of (C.2) by choosing a large C such that $u < \frac{1}{2}u^{\mathrm{T}} I(\boldsymbol{\beta}_0)u$.

Finally, note that

$$n\sum_{j=1}^{s}\Big\{\alpha_n P^{'}_{\lambda_n}(|\beta_{j0}|) sgn(\beta_{j0}) u_j + \frac{1}{2}\alpha_n^2 u_j^2 P^{''}_{\lambda_n}(|\beta_{j0}|)\{1+o(1)\}\Big\} \leq \sqrt{s} n\alpha_n a_n ||u|| + \frac{1}{2} n\alpha_n^2 ||u||^2 b_n. \tag{C.3}$$

Moreover, $\alpha_n = O_{\mathrm{p}}(n^{-\frac{1}{2}})$, then $n\alpha_n a_n = o_{\mathrm{p}}(n\alpha_n^2)$ provided that $a_n = o_{\mathrm{p}}(n^{-\frac{1}{2}})$. On the other hand, assume that $b_n = o_{\mathrm{p}}(1)$, we obtain $n\alpha_n^2 b_n = o_{\mathrm{p}}(n\alpha_n^2)$, then the order of the third term on the right-hand side of (C.2) is $o_{\mathrm{p}}(n\alpha_n^2)$. Hence, the order of (C.2) is $O_{\mathrm{p}}(n\alpha_n^2)$ and $D_n(u) < 0$ which implies $P\{\sup_{||u||=c} Q(\boldsymbol{\beta}_0+\alpha_n u) < Q(\boldsymbol{\beta}_0)\} \geq 1-\epsilon$ established by choosing a large C, this completes the proof of Theorem 1.

We then prove the theorem 2. First, we prove the sparsity of the parameters $\hat{\boldsymbol{\beta}}$.

(a) For any $\boldsymbol{\beta}_1$ satisfying $||\boldsymbol{\beta}_1-\boldsymbol{\beta}_{10}|| = O_{\mathrm{p}}(n^{-\frac{1}{2}})$, by the Taylor's expansion, we have

$$\begin{aligned} \frac{\partial Q(\boldsymbol{\beta})}{\partial\beta_j} &= \frac{\partial L(\boldsymbol{\beta})}{\partial\beta_j} - nP^{'}_{\lambda_n}(|\beta_j|) sgn(\beta_j) \\ &= \frac{\partial L(\boldsymbol{\beta}_0)}{\partial\beta_j} + \sum_{l=1}^{d}\frac{\partial^2 L(\boldsymbol{\beta}_0)}{\partial\beta_j\partial\beta_l}(\beta_l-\beta_{l0}) \\ &\quad + \sum_{l=1}^{d}\sum_{k=1}^{d}\frac{\partial^3 L(\boldsymbol{\beta}_*)}{\partial\beta_j\partial\beta_l\partial\beta_k}(\beta_l-\beta_{l0})(\beta_k-\beta_{k0}) \\ &\quad - nP^{'}_{\lambda_n}(|\beta_j|) sgn(\beta_j). \end{aligned} \tag{C.4}$$

Based on lemma 1, first, $E(\frac{\partial L(\boldsymbol{\beta}_0)}{\partial\beta_j}) = 0$, then $\frac{1}{n}\frac{\partial L(\boldsymbol{\beta}_0)}{\beta_j} = Op(n^{-\frac{1}{2}})$. Second,

$\frac{1}{n}\frac{\partial^2 L(\boldsymbol{\beta}_0)}{\partial\beta_j\partial\beta_l} = E(\frac{\partial^2 L(\boldsymbol{\beta}_0)}{\partial\beta_j\partial\beta_l}) + op\{1\}$. Hence,

$$\begin{aligned}\frac{\partial Q(\boldsymbol{\beta})}{\partial\beta_j} &= n\lambda_n\Big[\frac{1}{\lambda_n}\frac{1}{n}\frac{\partial L(\boldsymbol{\beta}_0)}{\partial\beta_j} + \frac{1}{\lambda_n}\frac{1}{n}\sum_{l=1}^{d}\frac{\partial^2 L(\boldsymbol{\beta}_0)}{\partial\beta_j\partial\beta_l}(\beta_l - \beta_l 0)\\ &\quad + \frac{1}{\lambda_n}\frac{1}{n}\sum_{l=1}^{d}\sum_{k=1}^{d}\frac{\partial^3 L(\boldsymbol{\beta}_*)}{\partial\beta_j\partial\beta_l\partial\beta_k}(\beta_l - \beta_{l0})(\beta_k - \beta_{k0}) - \frac{P'_{\lambda_n}(|\beta_j|)}{\lambda_n}sgn(\beta_j)\Big]\\ &= n\lambda_n\Big[\frac{1}{\lambda_n}O_{\mathrm{p}}(n^{-\frac{1}{2}})O_{\mathrm{p}}(n^{\frac{1}{4}}) + \frac{1}{\lambda_n}O_{\mathrm{p}}(n^{\frac{1}{2}})o_{\mathrm{p}}(n^0)O_{\mathrm{p}}(n^{-\frac{1}{2}})\\ &\quad + \frac{1}{\lambda_n}nO_{\mathrm{p}}(n^{\frac{1}{4}})O_{\mathrm{p}}(n^{\frac{1}{2}})O_{\mathrm{p}}(n^{-\frac{1}{2}})o_{\mathrm{p}}(n^0)O_{\mathrm{p}}(n^{-\frac{1}{2}})O_{\mathrm{p}}(n^{-\frac{1}{2}}) - \frac{P'_{\lambda_n}(|\beta_j|)}{\lambda_n}sgn(\beta_j)\Big]\\ &= n\lambda_n\Big[\frac{1}{\lambda_n}O_{\mathrm{p}}(n^{-\frac{1}{4}}) - \frac{P'_{\lambda_n}(|\beta_j|)}{\lambda_n}sgn(\beta_j)\Big].\end{aligned} \tag{C.5}$$

Because $n^{\frac{1}{4}}\lambda_n \to \infty$ when $n \to \infty$ and $\lambda_n \to 0$ and then $\frac{1}{\lambda_n}O_{\mathrm{p}}(n^{-\frac{1}{4}}) \to 0$. Moreover, $P_{\lambda_n}(|\beta_j|) \in \mathcal{P}$, then $P'_{\lambda_n}(0^+) \in (0,\infty)$ and $P'_{\lambda_n}(|\beta_j|) > 0$. In summary, the sign of $\frac{\partial Q(\boldsymbol{\beta})}{\partial\beta_j}$ depends only on β_j, which means

$$\frac{\partial Q(\boldsymbol{\beta})}{\partial\beta_j} < 0,\ for\ 0 < \beta_j < \alpha_n C, \tag{C.6}$$

and

$$\frac{\partial Q(\boldsymbol{\beta})}{\partial\beta_j} > 0,\ for\ -\alpha_n C < \beta_j < 0. \tag{C.7}$$

It is equivalent to say when $\boldsymbol{\beta}_2 = 0$, $\frac{\partial Q(\boldsymbol{\beta})}{\partial\beta_j} = 0$ and the maximum of $Q\begin{pmatrix}\boldsymbol{\beta}_1\\ \boldsymbol{\beta}_2\end{pmatrix}$ is $Q\begin{pmatrix}\boldsymbol{\beta}_1\\ 0\end{pmatrix}$ which means $\hat{\boldsymbol{\beta}}$ in Theorem 1 is a local maximizer.

Furthermore, we need to obtain the asymptotic normality of the parameter $\hat{\boldsymbol{\beta}}$, as shown in the following proofs.

(b) $\hat{\boldsymbol{\beta}}$ is a local maximizer and $\frac{\partial Q(\boldsymbol{\beta})}{\partial\hat{\beta}_j} = 0,\ j = 1, 2, ..., s$. Moreover,

$$\begin{aligned}\frac{\partial Q(\boldsymbol{\beta})}{\partial\hat{\beta}_j} &= \frac{\partial L(\boldsymbol{\beta})}{\partial\hat{\beta}_j} - nP'_{\lambda_n}(|\hat{\beta}_j|)sgn(\hat{\beta}_j)\\ &= \frac{\partial L(\boldsymbol{\beta}_0)}{\partial\hat{\beta}_j} + \sum_{l=1}^{s}\Big\{\frac{\partial^2 L(\boldsymbol{\beta}_0)}{\partial\beta_j\partial\beta_l} + o_{\mathrm{p}}(1)\Big\}(\hat{\beta}_l - \beta_{l0})\\ &\quad - n\Big[P'_{\lambda_n}(|\beta_{j0}|)sgn(\beta_{j0}) + \{P''_{\lambda_n}(|\beta_{j0}|) + op(1)\}(\hat{\beta}_j - \beta_{j0})\Big].\end{aligned} \tag{C.8}$$

Because $\hat{\boldsymbol{\beta}}_2 = 0$, thus the above formula$=L'(\boldsymbol{\beta}_{10}) - nI_1(\boldsymbol{\beta}_{10})(\hat{\boldsymbol{\beta}}_1 - \boldsymbol{\beta}_{10}) - nb - n\Sigma(\hat{\boldsymbol{\beta}}_1 - \boldsymbol{\beta}_{10})$. Hence, $L'(\boldsymbol{\beta}_{10}) = nI_1(\boldsymbol{\beta}_{10})(\hat{\boldsymbol{\beta}}_1 - \boldsymbol{\beta}_{10}) + nb + n\Sigma(\hat{\boldsymbol{\beta}}_1 - \boldsymbol{\beta}_{10})$ and both sides are divided by $\sqrt{n}$, we get

$$\frac{L'(\boldsymbol{\beta}_{10})}{\sqrt{n}} = \sqrt{n}(I_1(\boldsymbol{\beta}_{10}) + \Sigma)\Big\{\hat{\boldsymbol{\beta}}_1 - \boldsymbol{\beta}_{10} + (I_1(\boldsymbol{\beta}_{10}) + \Sigma)^{-1}b\Big\}. \tag{C.9}$$

From central limit theorem, $D\Big(\frac{L'(\boldsymbol{\beta}_{10})}{\sqrt{n}}\Big) = \frac{1}{n}E\Big(L'(\boldsymbol{\beta}_{10})L'(\boldsymbol{\beta}_{10})^{\mathrm{T}}\Big) = \frac{1}{n}I_1(\boldsymbol{\beta}_{10})$ and

$E\Big(\frac{L^{'}(\boldsymbol{\beta}_{10})}{\sqrt{n}}\Big) = 0$ which means

$$\frac{\frac{L^{'}(\boldsymbol{\beta}_{10})}{\sqrt{n}} - 0}{\sqrt{n}} \to \; N(0, \frac{1}{n} I_1(\boldsymbol{\beta}_{10})). \tag{C.10}$$

Hence, we conclude

$$\frac{L^{'}(\boldsymbol{\beta}_{10})}{\sqrt{n}} \to \; N(0, I_1(\boldsymbol{\beta}_{10})). \tag{C.11}$$

The above formula is equivalent to

$$\sqrt{n}(I_1(\boldsymbol{\beta}_{10}) + \Sigma)\Big\{\hat{\boldsymbol{\beta}}_1 - \boldsymbol{\beta}_{10} + (I_1(\boldsymbol{\beta}_{10}) + \Sigma)^{-1} b\Big\} \to \; N(0, I_1(\boldsymbol{\beta}_{10})). \tag{C.12}$$

Further, the asymptotic variance of $\hat{\boldsymbol{\beta}}_1$ is

$$\frac{1}{n}(I_1(\boldsymbol{\beta}_{10}) + \Sigma)^{-1} I_1(\boldsymbol{\beta}_{10})(I_1(\boldsymbol{\beta}_{10}) + \Sigma)^{-1}. \tag{C.13}$$

This completes the proof of Theorem 2. □

S4: Proof of Theorem 3

Proof. We obtain the bound of the risk function between our model and the Bayes classifier in this section. Before that, we give Assumption 1, Lemma 3 shows the bound of the model $R_{our}(x)$ and the Bayes classifier $\widetilde{R}^*(x)$ under the noisy labels.

Denote two events by $\{C(x) = 0\}$ and $\{C(x) = 1\}$, then $\mathbb{1}\{C(x) = 0\} + \mathbb{1}\{C(x) = 1\} = 1$.

Hence, we have

$$\begin{aligned}
R_{our}(x) &= \int_{\mathcal{X}} \mathbb{P}(Y_0 \neq C(x)|X = x) d\mathbb{P}_X(x) \\
&= \int_{\mathcal{X}} \Big\{ \mathbb{1}\{C(x) = 0\}\mathbb{P}(Y_0 \neq C(x)|X = x) \\
&\qquad + \mathbb{1}\{C(x) = 1\}\mathbb{P}(Y_0 \neq C(x)|X = x)\Big\} d\mathbb{P}_X(x) \\
&= \int_{\mathcal{X}} \Big\{ \mathbb{P}(C(x) = 0)\mathbb{P}(Y_0 = 1|X = x) \\
&\qquad + \mathbb{P}(C(x) = 1)\mathbb{P}(Y_0 = 0|X = x)\Big\} d\mathbb{P}_X(x) \\
&= \int_{\mathcal{X}} \Big\{ (1 - \eta(x)) + (2\eta(x) - 1)\mathbb{P}(C(x) = 0)\Big\} d\mathbb{P}_X(x).
\end{aligned} \tag{D.1}$$

Similarly,

$$\widetilde{R}^*(x) = \int_{\mathcal{X}} \Big\{ (1 - \eta(x)) + (2\eta(x) - 1)\mathbb{1}\{\widetilde{C}^*(x) < \frac{1}{2}\}\Big\} d\mathbb{P}_X(x). \tag{D.2}$$

For $\mathbb{P}_X$-almost all $x \in \mathcal{D}$,

$$\begin{aligned}
&\Big\{\mathbb{P}(C(x) = 0) - \mathbb{1}\{\widetilde{C}^*(x) < \frac{1}{2}\}\Big\}(2\eta(x) - 1) \\
&= \Big|\mathbb{P}(C(x) = 0) - \mathbb{1}\{\widetilde{C}^*(x) < \frac{1}{2}\}\Big| |2\eta(x) - 1| \\
&\leq \Big|\mathbb{P}(C(x) = 0) - \mathbb{1}\{\widetilde{C}^*(x) < \frac{1}{2}\}\Big| \\
&= \mathbb{P}(C(x) \neq \widetilde{C}^*(x)).
\end{aligned} \tag{D.3}$$

Moreover, for $\mathbb{P}_X$-almost all $x \in \mathcal{D}^c$, we have

$$\Big\{\mathbb{P}(C(x)=0) - \mathbb{1}\{\widetilde{C}^*(x) < \frac{1}{2}\}\Big\}(2\eta(x)-1) \leq 0. \tag{D.4}$$

It follows that

$$\begin{aligned}
R_{our}(x) - \widetilde{R}^*(x) &= \int_{\mathcal{X}} \Big\{\mathbb{P}(C(x)=0) - \mathbb{1}\{\widetilde{C}^*(x) < \frac{1}{2}\}\Big\}(2\eta(x)-1)d\mathbb{P}_X(x) \\
&= \int_{\mathcal{D}} \Big\{\mathbb{P}(C(x)=0) - \mathbb{1}\{\widetilde{C}^*(x) < \frac{1}{2}\}\Big\}(2\eta(x)-1)d\mathbb{P}_X(x) \\
&\quad + \int_{\mathcal{D}^c} \Big\{\mathbb{P}(C(x)=0) - \mathbb{1}\{\widetilde{C}^*(x) < \frac{1}{2}\}\Big\}(2\eta(x)-1)d\mathbb{P}_X(x) \\
&\quad + \int_{\mathcal{S}} \Big\{\mathbb{P}(C(x)=0) - \mathbb{1}\{\widetilde{C}^*(x) < \frac{1}{2}\}\Big\}(2\eta(x)-1)d\mathbb{P}_X(x) \\
&\to \int_{\mathcal{D}} \Big\{\mathbb{P}(C(x)=0) - \mathbb{1}\{\widetilde{C}^*(x) < \frac{1}{2}\}\Big\}(2\eta(x)-1)d\mathbb{P}_X(x) \\
&\quad + \int_{\mathcal{D}^c} \Big\{\mathbb{P}(C(x)=0) - \mathbb{1}\{\widetilde{C}^*(x) < \frac{1}{2}\}\Big\}(2\eta(x)-1)d\mathbb{P}_X(x) \\
&\leq \int_{\mathcal{D}} \Big\{\mathbb{P}(C(x)=0) - \mathbb{1}\{\widetilde{C}^*(x) < \frac{1}{2}\}\Big\}(2\eta(x)-1)d\mathbb{P}_X(x) \qquad \text{(D.5)} \\
&\leq \mathbb{P}\Big(\big\{C(x) \neq \widetilde{C}^*(x)\big\} \cap \{x \in \mathcal{D}\}\Big) \\
&\leq \mathbb{P}(C(x) \neq \widetilde{C}^*(x)) \\
&= \mathbb{P}(C(x)=1, \widetilde{C}^*(x)=0) + \mathbb{P}(C(x)=0, \widetilde{C}^*(x)=1) \\
&= \mathbb{P}(f(x) \geq \frac{1}{2}, \widetilde{\eta}(x) < \frac{1}{2}) + \mathbb{P}(f(x) < \frac{1}{2}, \widetilde{\eta}(x) \geq \frac{1}{2}) \\
&\leq \mathbb{P}(\widetilde{\eta}(x) + \epsilon \geq \frac{1}{2}, \widetilde{\eta}(x) < \frac{1}{2}) + \mathbb{P}(\widetilde{\eta}(x) - \epsilon < \frac{1}{2}, \widetilde{\eta}(x) \geq \frac{1}{2}) \\
&= \mathbb{P}(\frac{1}{2} - \epsilon \leq \widetilde{\eta}(x) < \frac{1}{2}) + \mathbb{P}(\frac{1}{2} \leq \widetilde{\eta}(x) < \frac{1}{2} + \epsilon) \\
&= \mathbb{P}(\frac{1}{2} - \epsilon \leq \widetilde{\eta}(x) < \frac{1}{2} + \epsilon) \\
&\leq M\{O(\epsilon)\}^{\lambda},
\end{aligned}$$

by (D.3), (D.4) and $t \leq t_0 \to 0$. This completes the proof of Lemma 3.

And then, we can prove our main result. Based on Lemma 3, we have

$$\begin{aligned}
R_{our}(x) - R^*(x) &= \int_{\mathcal{X}} \Big\{\mathbb{P}(C(x)=0) - \mathbb{1}\{C^*(x) < \frac{1}{2}\}\Big\}(2\eta(x)-1)d\mathbb{P}_X(x) \\
&= \int_{\mathcal{X}} \Big\{\mathbb{P}(C(x)=0) - \mathbb{1}\{\widetilde{C}^*(x) < \frac{1}{2}\}\Big\}(2\eta(x)-1)d\mathbb{P}_X(x) \\
&\quad + \int_{\mathcal{X}} \Big\{\mathbb{1}\{\widetilde{C}^*(x) < \frac{1}{2}\} - \mathbb{1}\{C^*(x) < \frac{1}{2}\}\Big\}(2\eta(x)-1)d\mathbb{P}_X(x) \\
&= R_{our}(x) - \widetilde{R}^*(x) \\
&\quad + \int_{\mathcal{X}} \Big\{\mathbb{1}\{\widetilde{C}^*(x) < \frac{1}{2}\} - \mathbb{1}\{C^*(x) < \frac{1}{2}\}\Big\}(2\eta(x)-1)d\mathbb{P}_X(x) \\
&\leq M\{O(\epsilon)\}^{\lambda} \\
&\quad + \int_{\mathcal{X}} \Big\{\mathbb{1}\{\widetilde{C}^*(x) < \frac{1}{2}\} - \mathbb{1}\{C^*(x) < \frac{1}{2}\}\Big\}(2\eta(x)-1)d\mathbb{P}_X(x)
\end{aligned}$$

$$
\begin{aligned}
&= M\{O(\epsilon)\}^{\lambda} + \int_{\mathcal{D}} \Big\{ \mathbb{1}\{\widetilde{C}^*(x) < \frac{1}{2}\} - \mathbb{1}\{C^*(x) < \frac{1}{2}\} \Big\} (2\eta(x) - 1) d\mathbb{P}_X(x) \\
&\quad + \int_{\mathcal{D}^c} \Big\{ \mathbb{1}\{\widetilde{C}^*(x) < \frac{1}{2}\} - \mathbb{1}\{C^*(x) < \frac{1}{2}\} \Big\} (2\eta(x) - 1) d\mathbb{P}_X(x) \\
&\quad + \int_{\mathcal{S}} \Big\{ \mathbb{1}\{\widetilde{C}^*(x) < \frac{1}{2}\} - \mathbb{1}\{C^*(x) < \frac{1}{2}\} \Big\} (2\eta(x) - 1) d\mathbb{P}_X(x) \\
&\to M\{O(\epsilon)\}^{\lambda} + \int_{\mathcal{D}} \Big\{ \mathbb{1}\{\widetilde{C}^*(x) < \frac{1}{2}\} - \mathbb{1}\{C^*(x) < \frac{1}{2}\} \Big\} (2\eta(x) - 1) d\mathbb{P}_X(x) \\
&\quad + \int_{\mathcal{D}^c} \Big\{ \mathbb{1}\{\widetilde{C}^*(x) < \frac{1}{2}\} - \mathbb{1}\{C^*(x) < \frac{1}{2}\} \Big\} (2\eta(x) - 1) d\mathbb{P}_X(x) \\
&= M\{O(\epsilon)\}^{\lambda} + \mathbb{P}\Big(\big\{ C^*(x) \neq \widetilde{C}^*(x) \big\} \cap \{ x \in \mathcal{D}^c \} \Big) \qquad (D.6) \\
&\leq M\{O(\epsilon)\}^{\lambda} + \mathbb{P}\Big(C^*(x) \neq \widetilde{C}^*(x) \Big) \\
&= M\{O(\epsilon)\}^{\lambda} + \mathbb{P}\Big(C^*(x) = 1, \widetilde{C}^*(x) = 0 \Big) + \mathbb{P}\Big(C^*(x) = 0, \widetilde{C}^*(x) = 1 \Big) \\
&= M\{O(\epsilon)\}^{\lambda} + \mathbb{P}\Big(\eta(x) \geq \frac{1}{2}, \widetilde{\eta}(x) < \frac{1}{2} \Big) + \mathbb{P}\Big(\eta(x) < \frac{1}{2}, \widetilde{\eta}(x) > \frac{1}{2} \Big) \\
&= M\{O(\epsilon)\}^{\lambda} + \mathbb{P}\Big(\eta(x) \geq \frac{1}{2}, \eta(x) < \frac{\frac{1}{2} - \tau_{01}(x)}{1 - \tau_{01}(x) - \tau_{10}(x)} \Big) \\
&\quad + \mathbb{P}\Big(\eta(x) < \frac{1}{2}, \eta(x) \geq \frac{\frac{1}{2} - \tau_{01}(x)}{1 - \tau_{01}(x) - \tau_{10}(x)} \Big).
\end{aligned}
$$

If $\tau_{01}(x) \leq \tau_{10}(x)$,

$$
\begin{aligned}
(D.6) &= M\{O(\epsilon)\}^{\lambda} + \mathbb{P}\Big(\eta(x) \geq \frac{1}{2}, \eta(x) < \frac{\frac{1}{2} - \tau_{01}(x)}{1 - \tau_{01}(x) - \tau_{10}(x)} \Big) \\
&\leq M\{O(\epsilon)\}^{\lambda} + M\Big\{ \frac{\frac{1}{2}(\tau_{10}(x) - \tau_{01}(x))}{1 - \tau_{01}(x) - \tau_{10}(x)} \Big\}^{\lambda} \qquad (D.7) \\
&= M\{O(\epsilon)\}^{\lambda} + K(\lambda).
\end{aligned}
$$

If $\tau_{01}(x) > \tau_{10}(x)$,

$$
\begin{aligned}
(D.6) &= M\{O(\epsilon)\}^{\lambda} + \mathbb{P}\Big(\eta(x) < \frac{1}{2}, \eta(x) \geq \frac{\frac{1}{2} - \tau_{01}(x)}{1 - \tau_{01}(x) - \tau_{10}(x)} \Big) \\
&\leq M\{O(\epsilon)\}^{\lambda} + M\Big\{ \frac{\frac{1}{2}(\tau_{10}(x) - \tau_{01}(x))}{1 - \tau_{01}(x) - \tau_{10}(x)} \Big\}^{\lambda} \qquad (D.8) \\
&= M\{O(\epsilon)\}^{\lambda} + K(\lambda).
\end{aligned}
$$

And this completes the proof of Theorem 3. □

S5: Proof of Theorem 4

Proof. We prove the convergence using the difference of the objective function values and Taylor expansion in two consecutive iterations,

$$
\begin{aligned}
Q(\boldsymbol{\beta}_{t+1}) - Q(\boldsymbol{\beta}_t) &\leq \nabla Q(\boldsymbol{\beta}_t)^{\mathrm{T}}(\boldsymbol{\beta}_{t+1} - \boldsymbol{\beta}_t) + \frac{L}{2}|\boldsymbol{\beta}_{t+1} - \boldsymbol{\beta}_t|^2 \\
&= \nabla Q(\boldsymbol{\beta}_t)^{\mathrm{T}}\Big\{ - \eta\Big(N_{non-noisy} - N_{noisy}\Big)\nabla Q(\boldsymbol{\beta}_t)\Big\} \\
&\quad + \frac{L}{2}| - \eta\Big(N_{non-noisy} - N_{noisy}\Big)\nabla Q(\boldsymbol{\beta}_t)|^2 \\
&= -\eta\Big(N_{non-noisy} - N_{noisy}\Big)|\nabla Q(\boldsymbol{\beta}_t)|^2 \\
&\quad + \frac{L\eta^2}{2}\Big(N_{non-noisy} - N_{noisy}\Big)^2|\nabla Q(\boldsymbol{\beta}_t)|^2 \\
&= -\eta\Big(N_{non-noisy} - N_{noisy}\Big)\Big\{1 - \frac{L\eta}{2}\Big(N_{non-noisy} - N_{noisy}\Big)\Big\}|\nabla Q(\boldsymbol{\beta}_t)|^2 \\
&\leq 0.
\end{aligned}
\tag{E.1}
$$

The last equation is obatined by $N_{non-noisy} > N_{noisy}$. We obtain the function values $Q(\boldsymbol{\beta}_t)$ form a non-increasing sequence, which implies that $Q(\boldsymbol{\beta}_t)$ converges. And this completes the proof of Theorem 4. □

References

1. Babenko V, Babenko Y, Kriachko N, Skorokhodov D. On Hardy-Littlewood-Pólya and Taikov type inequalities for multiple operators in Hilbert spaces. Analysis Mathematica. 2021;47:709–745.
